# Abdominal wall hernia repair: from prosthetic meshes to smart materials

**DOI:** 10.1016/j.mtbio.2023.100691

**Published:** 2023-06-29

**Authors:** Qimanguli Saiding, Yiyao Chen, Juan Wang, Catarina Leite Pereira, Bruno Sarmento, Wenguo Cui, Xinliang Chen

**Affiliations:** a Shanghai Key Laboratory of Embryo Original Diseases, The International Peace Maternal and Child Health Hospital, Shanghai Jiao Tong University School of Medicine, 910 Hengshan Road, Shanghai, 200030, PR China; b Department of Orthopaedics, Shanghai Key Laboratory for Prevention and Treatment of Bone and Joint Diseases, Shanghai Institute of Traumatology and Orthopaedics, Ruijin Hospital, Shanghai Jiao Tong University School of Medicine, 197 Ruijin 2nd Road, Shanghai, 200025, PR China; c I3S – Instituto de Investigação e Inovação Em Saúde and INEB – Instituto de Engenharia Biomédica, Universidade Do Porto, Rua Alfredo Allen 208, 4200-135, Porto, Portugal; d IUCS – Instituto Universitário de Ciências da Saúde, CESPU, Rua Central de Gandra 1317, 4585-116, Gandra, Portugal

**Keywords:** Abdominal wall hernia, Biological tissue grafts, Electrospinning, Hydrogel scaffold, Polypropylene mesh

## Abstract

Hernia reconstruction is one of the most frequently practiced surgical procedures worldwide. Plastic surgery plays a pivotal role in reestablishing desired abdominal wall structure and function without the drawbacks traditionally associated with general surgery as excessive tension, postoperative pain, poor repair outcomes, and frequent recurrence. Surgical meshes have been the preferential choice for abdominal wall hernia repair to achieve the physical integrity and equivalent components of musculofascial layers. Despite the relevant progress in recent years, there are still unsolved challenges in surgical mesh design and complication settlement. This review provides a systemic summary of the hernia surgical mesh development deeply related to abdominal wall hernia pathology and classification. Commercial meshes, the first-generation prosthetic materials, and the most commonly used repair materials in the clinic are described in detail, addressing constrain side effects and rational strategies to establish characteristics of ideal hernia repair meshes. The engineered prosthetics are defined as a transit to the biomimetic smart hernia repair scaffolds with specific advantages and disadvantages, including hydrogel scaffolds, electrospinning membranes, and three-dimensional patches. Lastly, this review critically outlines the future research direction for successful hernia repair solutions by combing state-of-the-art techniques and materials.

## Graphical abstract

**Figure:**
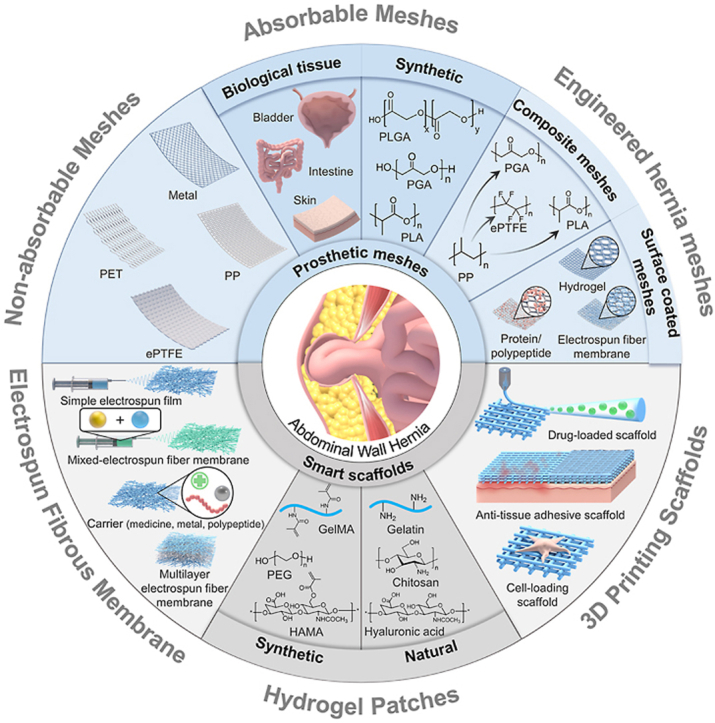

## 1 Introduction

The human abdominal wall is a well-structured construction of skin, subcutaneous fat tissues, several muscle layers, preperitoneal fascia, and peritoneum that attach to each other and the bone to hold the abdominal cavity contents. It has a pivotal role in the protection of postural assistance and abdominal pressure stabilization [1,2]. An abdominal hernia can occur when weak, defective, and injured areas in the abdominal wall allowing the protrude of abdominal organs of part of any organ [3]. Both endogenous (gene, age, gender) and exogenous (drug, smoking, and trauma) factors are behind the abdominal wall hernia development.

Epidemiological datasets show that hernia repair brings a tremendous economic burden to countries annually [4]. Anatomically, abdominal hernias can be divided into ventral, parastomal, umbilical, and Spigelian hernias, as these parts are vulnerable to congenital defects, external trauma, and abdominal surgeries [[5], [6], [7]]. Abdominal hernias are most commonly diagnosed as pain with an irregular protuberance in the abdominal wall, still with low morbidity and mortality [8]. However, when the herniated abdominal viscera are incarcerated, ischemia and death of the involved organs can happen if not promptly treated [9,10].

Clinically, surgeons usually strengthen the defect and restore the normal anatomical structure with sutures or surgical meshes [11]. Direct suturing, however, usually results in postoperative chronic pain and a high recurrence rate due to excessive tension. Mesh repair, on the other hand, often fails because of severe complications such as visceral adhesions, mesh shrinkage-induced pain, bacterial infection, and intercourse difficulty, leading to revision surgery [[12], [13], [14]]. Researchers have proposed valid alternatives in the past decade to minimize mesh-related side effects. The modification of commercial meshes with biocompatible agents such as proteins, peptides, and even engineered hydrogels to improve hernia meshes' histocompatibility has been one of the most explored approaches [15,16]. Anticipated outcomes were achieved in the short term, but leaving the unsatisfying hernia repair owing to the low stability of these modification methods. In addition, some alternative solutions, such as xenograft transplantation [17], decellularized matrices [18,19], and small intestinal submucosa implantation [20,21], were also tried in the clinic. These approaches have also faced new issues related to poor mechanical strength, disease transfection, and donor site morbidity, limiting their widespread applications.

Looking through the hernia repair evolution history over the decades, an optimal approach should be designed with low risks of infection and visceral adhesion, high mechanical characteristics to support internal content, cost efficiency for broad applications, and easy handling properties to conveniently implant and fix to the abdominal wall. The urgent, yet unmet need, for patient-friendly and tissue-biocompatible repairing approaches then shifted to tissue engineering as it brought new insights into disease treatment and prevention.

Hydrogels, with simple composition [22,23] or chemically modified or physically doped, can produce adhesive and tough hydrogels, which can be used for tissue regeneration including hernia. Due to the relatively weak mechanical strength, hydrogels are used as surface coatings to reduce tissue adhesion or increase the biocompatibility of hernia repair materials [24,25]. Electrospinning, on the other hand, has emerged as a leading strategy in tissue engineering to fabricate fibrous membranes with large surface-area-to-volume ratios and nano-to-micro-sized fibers [26,27]. Owing to its diversity in composition, electrospun fibrous membranes have been investigated in various tissue engineering fields, such as cardiac patches [28], wound dresses [29], and drug-delivering carriers [26]. The ideal mechanical strength of the fibers is also one of the significant advantages of this technique, which made it an ideal mesh option for abdominal wall hernia reconstruction in the past decade [30,31]. However, due to the inertness of the row materials used for electrospinning, the obtained fibers are primarily hydrophobic, which will hinder their histological integration when applied in hernia repair. To provide the required biocompatibility and mechanical strength for hernia repair, researchers introduced 3D printed scaffolds into the abdominal wall hernia reconstruction [32,33]. The adjustable structural properties and a wide range of printing sources made 3D printing a future trend for designing smart hernia repair materials [34].

**Fig. 1:**
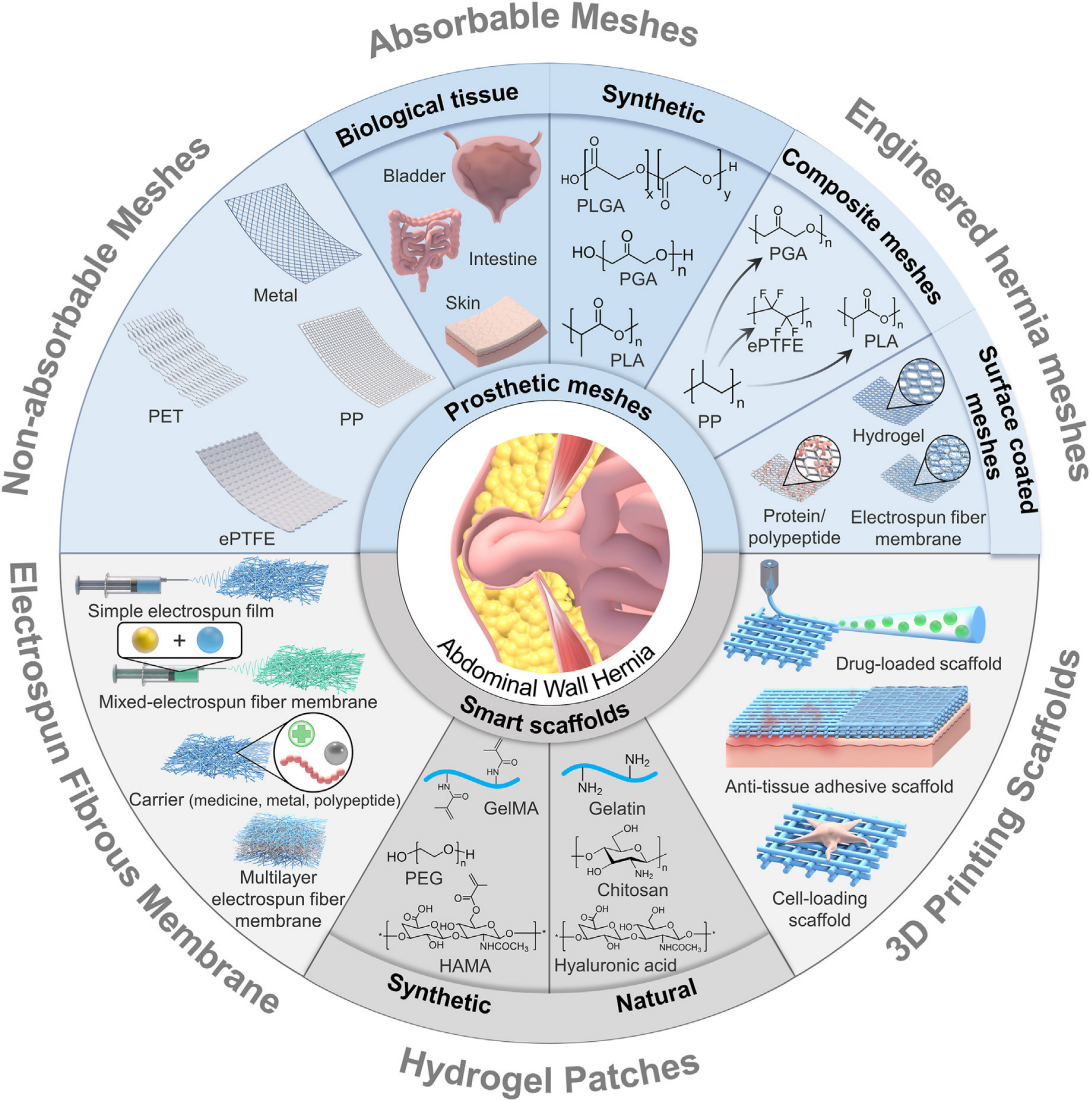
Schematic illustration of abdominal wall hernia repair meshes: from prosthetic meshes to smart materials.

This review aims to summarize and update, in a deep critical manner, on major evolutions in hernia repair material design and application. Abdominal wall anatomy and hernia pathogenesis is first shortly introduced, followed by the hernia classifications, clinical solutions, and the accompanying complications. Then, the revolution in commercial meshes is described. Next, the biological tissue grafts used for hernia repair are presented. Afterward, we highlighted the smart engineering materials, including hydrogels, electrospun fibrous membranes, and 3D scaffolds that have been investigated in recent years (Fig. 1). A rational conclusion is drawn with remarks on the challenges and future directions for fabrication and use of smart materials for abdominal hernia repair.

## 2 Abdominal hernia pathogenesis

### 2.1 Abdominal anatomy and hernia pathogenesis

The human abdominal wall is composed of skin, subcutaneous fat, several fascia layers, and interconnected muscle layers in proper sequence [35]. The exact anatomical structure of the abdominal wall differs depending on the location. Despite the complexity of the abdominal wall composition, multiple muscle groups work in conjunction to provide mechanical support for abdominal cavity contents and generate abdominal pressure to support physiologic functions such as laughing, lifting, coughing, and standing [36,37]. In addition to the strong muscle layers, there are nerves, blood vessels, and lymphatic vessels.

**Fig. 2:**
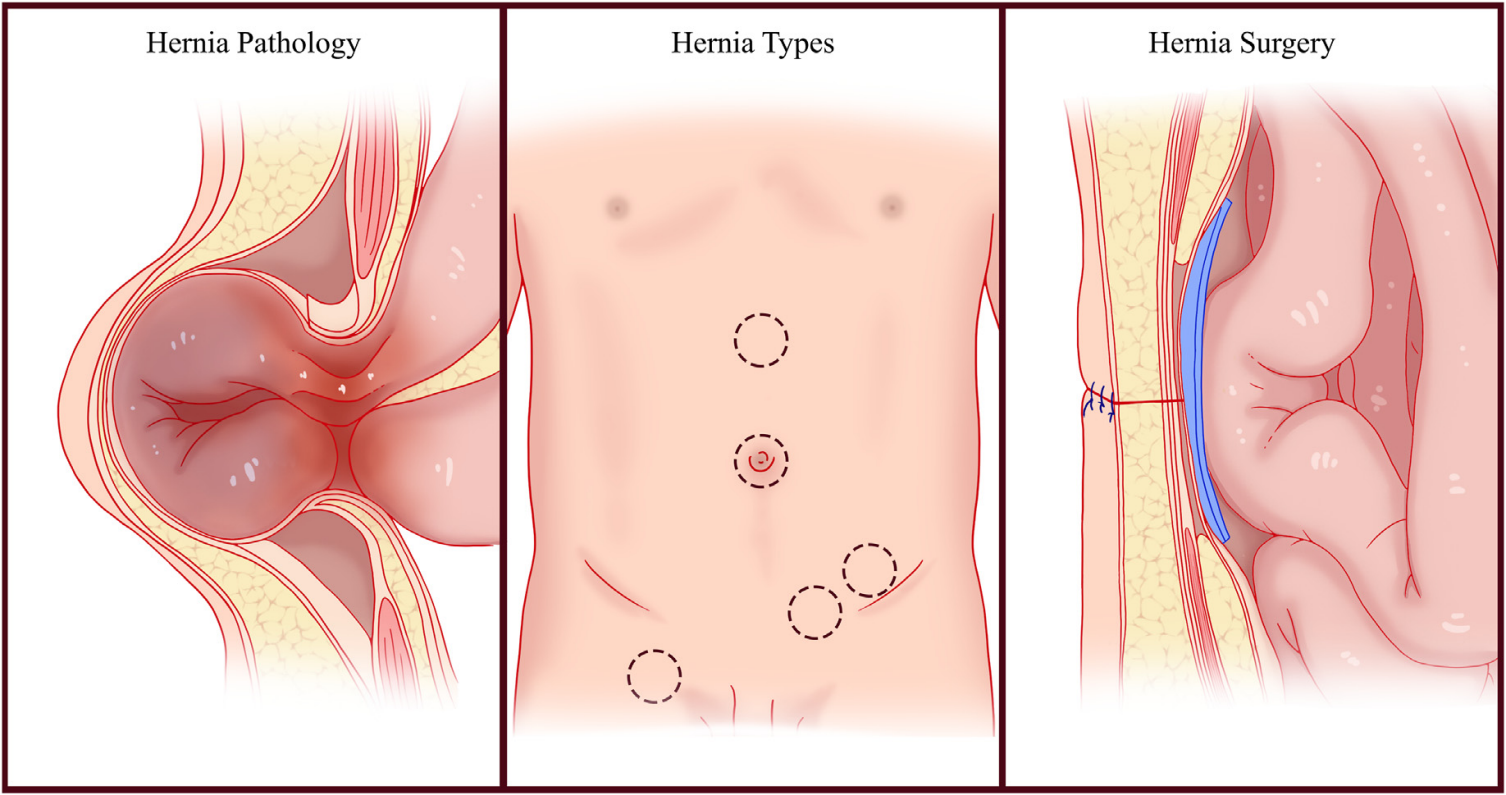
Schematic illustration of hernia pathology, hernia types and hernia surgery.

The structure integrity of human abdominal wall is primarily maintained by a set of muscles that provide sufficient mechanical strength and elasticity to hold the intraabdominal organs (Fig. 2). In general terms, an abdominal hernia is an abnormal protrusion of intra-abdominal contents resulting from a defect in the ability of the abdominal wall structural tissues to support the torso [38]. Abdominal hernias occur when the structural steadiness of the abdominal wall is disrupted by multifactorial processes, including endogenous factors such as age, gender, anatomic variations, and family histories and exogenous factors such as obesity, ascites, cigarette smoking, pregnancy, and surgery [39]. The biological foundations of the hernia remain to be unveiled despite scholars in recent decades proposing several molecular and cellular mechanisms [40,41]. Among them, abnormal extracellular matrix (ECM) metabolism, especially collagen metabolism, is an early target of investigations into the pathogenesis of hernia [42]. Apart from the decreased amount of collagen, the ratio abnormality of collagen isoforms (collagen I and collagen III) is also evident in both the primary and secondary hernias [43]. Growth factors [44], nutrition [45], and alterations in cell phenotypes [46] are possible mechanisms of hernia occurrence. Regarding the cellular population taking part in the process, fibroblasts are the most recognized cells that play critical roles in the ECM integrity and mechanical stretching of the abdominal wall [47]. Although the cellular composition during abdominal wound repairing is not completely understood, the rapid development of life science technologies, including single-cell RNA sequencing, has enabled the investigation of cell components that contribute to the pathological process. The transcriptome atlas of other types of hernias has shown that a wide variety of immune cells also take part in the pathophysiological process [48,49]. However, the more specific cell-cell interactions and gene expression landscapes of abdominal hernia remain unveiled.

### 2.2 Classification of abdominal hernia and treatment solutions

To compare different studies and publications on hernias and develop evidence-based therapeutic strategies, it is of great importance to make definitive classifications of abdominal wall hernia. According to the European Hernia Society classification system, the primary and incisional abdominal hernias are classified based on localization and size [50,51]. The specific hernia classifications are given in Table 1.

**Table 1:** The classification of abdominal wall hernias.

Classification	Localization		Size	
Primary Abdominal Wall Hernia	Midline	Epigastric	Diameter	Small: <2 ​cm; Medium: ≥2–4 ​cm; Large: ≥4 ​cm
		Umbilical		
	Lateral	Spigelian		
		Lumbar		
Incisional Abdominal Wall Hernia	Midline	Subxiphoidal	Width	W1：< 4 ​cm; W2: ≥4–10 ​cm; W3: ≥10 ​cm
		Epigastric		
		Umbilical		
		Infraumbilical		
		Suprapubic		
	Lateral	Subcostal		
		Flank		
		Iliac		
		Lumbar		

Despite the variety of abdominal hernias, the reported incidence of different types can differ. For instance, umbilical hernias are the most common ventral hernias, resulting from abnormal closure of abdominal wall defect as a primary hernia or a postoperative abdominal wall gap as an incisional hernia. This is a key anatomic landmark to precisely locate the hernia help making an accurate diagnosis for immediate treatment.

Surgery is the most common solution for abdominal wall hernia repair, and sutures or meshes can achieve hernia closure [52,53]. Suture repair is the earliest abdominal hernia repair strategy and aims to close abdominal defects with sutures under tension. Although the procedures used by early surgeons are effective, tension-induced pain and the high recurrence rate of suture techniques remain major challenges in suture repair [54,55]. Forced by the overall repair failure of suture techniques, metal sutures, plastics, and synthetic polymeric meshes are introduced into the clinic successively by the plastic surgeons. With the improvement of hernia plastics and surgeons’ skill level, the difficulty of restoring the integrity of abdominal wall have been gradually decreasing. But abdominal stiffness, tissue adhesion, and mesh erosion are new complications that need to be overcome by joint efforts by both surgeons and engineers in the near future. Quick development of the medical apparatus and bioresorbable implant science enabled tremendous progress forming a clear two-phase evolutionary process of synthetic meshes and smart biomaterials in hernia repair history. It is also the transitory process between clinical application and basic research.

## 3 Commercial meshes used in clinics for hernia repair

With the rapid development of health services, the progress of medical science and technology, and the improvements in materials research, new hernia repair meshes are constantly emerging. The surgeons choose the most reasonable meshes among the different options because the implant variables determine or affect treatment choice and the prognosis of hernia. Before the introduction of hernia materials, simple suture was first adopted by surgeons to close the abdominal wall. Hernia repair was first carried out with silk sutures, later transitioning to silver ones. There were other suture techniques such as fascial closure, fascial overlapping, and retention suture. Despite the certain successful rate, suturing under excessive tension brought about unbearable pain, suture rupture and ischemia in hernia patients with high recurrence rates. Given the unacceptable outcomes of suture operation, the tension-free repair concept had cut a striking figure as early as in the beginning of last century. The plastics became the leading role in hernia repair area. The early hernia meshes were classified into two categories from the prospective of degradation performance: non-absorbable and absorbable meshes (Table 3).

**Table 2:** The characteristic classification of prosthetic meshes.

Classification	Parameters
Filament Constitution	Monofilament
	Multifilament
Pore Size	Very Large pore: >2000 um
	Large Pore: 1000–2000 um
	Medium Pore: 600–1000 um
	Small Pore: 100–600 um
	Microporous: <100 um
Mesh Weight	Heavy Weight: >90 ​g/m^2^
	Medium Weight: 50–90 ​g/m^2^
	Light Weight: 35–50 ​g/m^2^
	Ultra-light Weight: <35 ​g/m^2^
Pore Shape	Square
	Diamond
	Hexagon

**Table 3:** The classification of commercial hernia meshes.

	Classification	Components	Brand	References
Non-absorbable Meshes	Metal	Silver, Steel, Tantalum	Goepel; Burke; Babcock	[56,57,58]
	Polymer	PET	Mersilene; Parietex	[59,60]
		ePTFE	DualMesh; MycroMesh;	[[61], [62], [63]]
		PP	Prolene; Parietene; Serapren; Marlex; Prolite;	[64,65,[66], [67], [68], [69], [70]]
Absorbable Meshes	Synthetic Single Polymer	PGA	Dexon; Safil	[71,72]
		PLGA	Vicryl;	[73,74,75]
		PLA	Polylactide Mesh	[76,77,78,79]
		P4HB	Phasix	[80,81]
		PVDF	Dynamesh	[82,83]
	Synthetic Composite Polymers	PLA/PTMC	TIGR	[84]
		PGA/PTMC	GORE BIOA	[85,86]
	Biological grafts	Small Intestinal Mucosa	Surgisis; Fortagen	Single SIS Patch [87,88,89,[90], [91], [92], [93], [94], [95], [96], [97], [98], [99], [100], [101]] Composite SIS Patch [102,103,[104], [105], [106], [107], [108], [109]]
		Acellular Dermal Matrix	Alloderm; Allomax; FlexHD; Permacol; Collamend; XenMatriX; SurgiMend	Human Dermal Matrix [[110], [111], [112],^113^,^114^,^115^,[116], [117], [118], [119], [120], [121], [122], [123], [124], [125], [126], [127]] Porcine Dermal Matrix [[128], [129], [130], [131],[132], [133], [134], [135], [136], [137], [138], [139], [140], [141], [142], [143], [144], [145]]
		Acellular Bladder Matrix	Gentrix	[146,[147], [148], [149], [150], [151], [152], [153]]
		Acellular Pericardial Patch	Veritas; Tutopas; Periguard	[[154], [155], [156],^157^,[158], [159], [160]]

### 3.1 Non-absorbable meshes

Hernia reconstructive surgery is one of the most commonly practiced surgical procedures per year, with consistently increasing inpatient costs. Prosthetic meshes combined with “tension-free hernioplasty” is the general approach for hernia repair [161,162]. The dynamic development of medical technologies forced the hernia material engineering which paved pathways for abdominal wall reconstruction with synthetic industrial products. Apart from the metal protheses, the other synthetic non-absorbable hernia meshes are still the most popular ones in clinic.

#### 3.1.1 Metal prostheses

Metal prosthesis is the earliest repair material used for hernia repair. In 1900, Witzel and Goepel used a silver mesh to repair hernia for the first time which was continuously practiced in clinic until 1960s [163]. However, the rigidity, poor tissue integration, and severe inflammatory reaction prompted its replacement by other metals such as tantalum. Tantalum mesh was used as hernia repair material, but it has been gradually discarded due to complications such as mesh contraction and rupture post implantation [56]. Babcock claimed that stainless steel was the best prosthetic material for abdominal hernia in 1952. Nevertheless, its rigidity led to abdominal stiffness, chronic pain and discomfort, as well as sinus formation [57]. Therefore, metal prostheses have gradually been replaced by plastic meshes.

#### 3.1.2 Polyester

Polyester (PET)-based prostheses were the first non-metallic approaches in abdominal hernia repair by Wolstenholme in 1956 [164] and have become a commercial product under the trade name “Mersilene”. Stoppa and colleagues applied PET prostheses in the peritoneal area to repair abdominal wall defects. Because there was no suture fixing in this approach, the hernia repair efficacy mostly relied on the tissue ingrowth around the implant [165]. The suppleness and hydrophilicity attracted most surgeons but its poor tensile strength, susceptibility to fibrosis, higher recurrence rate resulted in substantial reduction in clinical application. All the encountered complications made a transition to expanded polytetrafluoroethylene (ePTFE) based materials which was regarded to be more resistant to bacterial infection and sinus formation (REF missing?).

#### 3.1.3 Expanded polytetrafluoroethylene

Polytetrafluoroethylene (PTFE), first discovered in 1938, was a slippery and inert substance that made its way into herniology in 1959. But the extremely low mechanical strength and high recurrence rate became the reason for its abandonment. However, W.L Gore et al. refined the process to expand the Teflon into a porous structure with improved tensile strength and made a vascular prosthesis before introducing it into hernia repair field in 1980 [166]. Clinical results were better, causing less adhesions and infections. This type of mesh is typically represented by the MycroMesh and DualMesh produced by Gore Company in the United States. Specifically, surface aperture of DualMesh＜3 ​μm, which can prevent cell ingrowth and is particularly ideal incisional hernia repair. Compared with the knitted PET, the microporous structure of the ePTFE body gives it a better anti-adhesion effect. Simultaneously, however, bacteria can invade freely, while inflammatory cells such as macrophages are difficult to enter the mesh area, resulting in a poor anti-infection capability of ePTFE mesh, leading to its immediate removal. Besides, the high cost is also one important factor for finding another solution in hernioplasty.

#### 3.1.4 Polypropylene

**Fig. 3:**
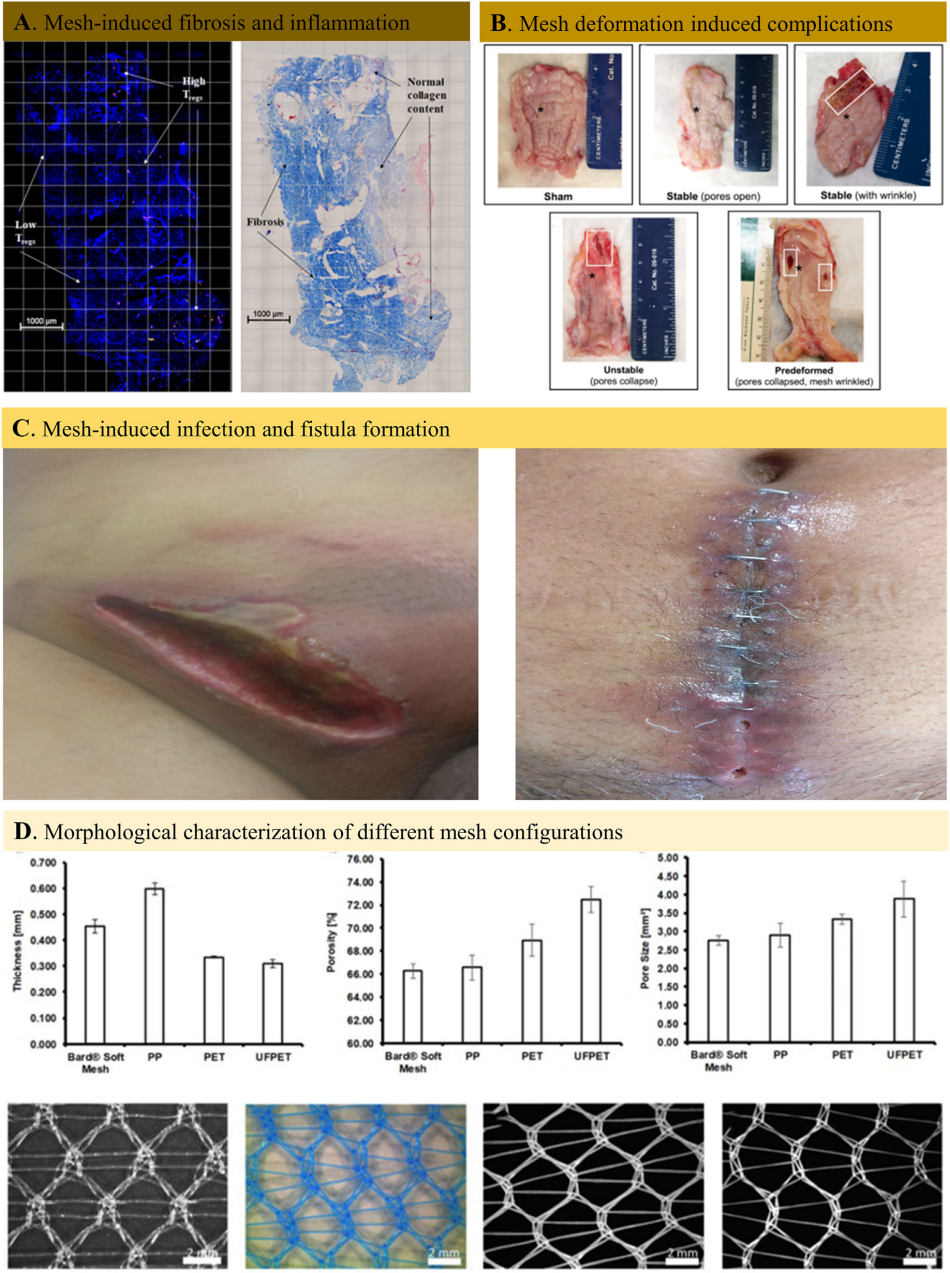
Non-absorbable hernia mesh-induced complications and structural difference between various commercial meshes. A. Immunofluorescent image of mesh-tissue explant (left) and Mosson's trichrome staining result of the same mesh-tissue explant with collagen deposition (right). The results demonstrated the inflammatory cell infiltration and fibrosis within the hernia mesh implant site [167]. B. Gross observation of the explanted mesh-vagina complexes and the implant complications related with hernia mesh deformation [64]. C. Post-operative case of infection and fistula formation after hernioplasty [65]. D. Morphological characterization (thickness, porosity, and pore size) and light microscopic pictures of different non-absorbable commercial meshes [168].

Hitherto, the polypropylene (PP) mesh was the most commonly used hernia prothesis in clinics. The American surgeon Usher first experimented the strong, inert, and non-wettable polymer in animal models to confirm its effectiveness for hernia repair as early as in 1950s. After being refined, the PP mesh was utilized in clinics several years later and strengthened the defect area by initial inflammation and the follow-up fibrosis but without encapsulation. The repair mechanism avoided most of the complications of the earlier hernia prostheses making the PP mesh become the best prosthetic hernia material. A number of technical innovations have been made along the decades to enhance its tissue integrability, anti-infectious property and chemical stability. Despite the excellent hernia repair outcomes of PP mesh, the multiple complications such as mesh-induced inflammation and fibrosis (Fig. 3A), foreign body reaction, mesh contraction (Fig. 3B), infection and fistula formation (Fig. 3C), chronic pain and peritoneal organ adhesion have pushed both the surgeons and engineers to rethink solution for successful hernia repair.

Most of the non-degradable polypropylene filaments knitted tightly together was inherently inert and inevitable to create a local inflammatory response, resulting in tissue fibrosis, scar formation, and mesh contraction. Despite many variables of interest for proposing a classification of the early prototype meshes (mainly PP, PET and ePTFE), the four critical parameters, including filament constitution, pore size, and mesh weight (Table 2), are adopted to propose a functional classification of non-absorbable hernia meshes. In most cases, the prototype meshes are classified under complex parameter combinations, for example, light-weight, very large pore, square knit pattern meshes (LWVLS), medium-weight, very large pore, square knit pattern meshes (MWVLS), light-weight, medium pore, diamond knit pattern meshes (LWMD), medium-weight, medium pore, diamond knit pattern meshes (MWMD), medium-weight, very large pore, hexagon knit pattern (MWVLH), etc. (Fig. 3D).

### 3.2 Absorbable meshes

The increasing need for novel hernia repair materials with the aim of reducing mesh-related complications require a thorough evaluation of the hernia meshes that are presently available in clinical use so as to create new surgical mesh alternatives. In this context, the application of absorbable meshes is a new trend in abdominal wall reconstruction surgery. Compared with non-absorbable, absorbable hernia meshes can be gradually absorbed by the host tissue after being implanted in the body. They can provide temporary scaffolds for tissue reconstruction posing a great potential for abdominal wall hernia repair. The absorbable meshes can be divided into synthetic or biological meshes according to the material source.

#### 3.2.1 Synthetic absorbable meshes

**Fig. 4:**
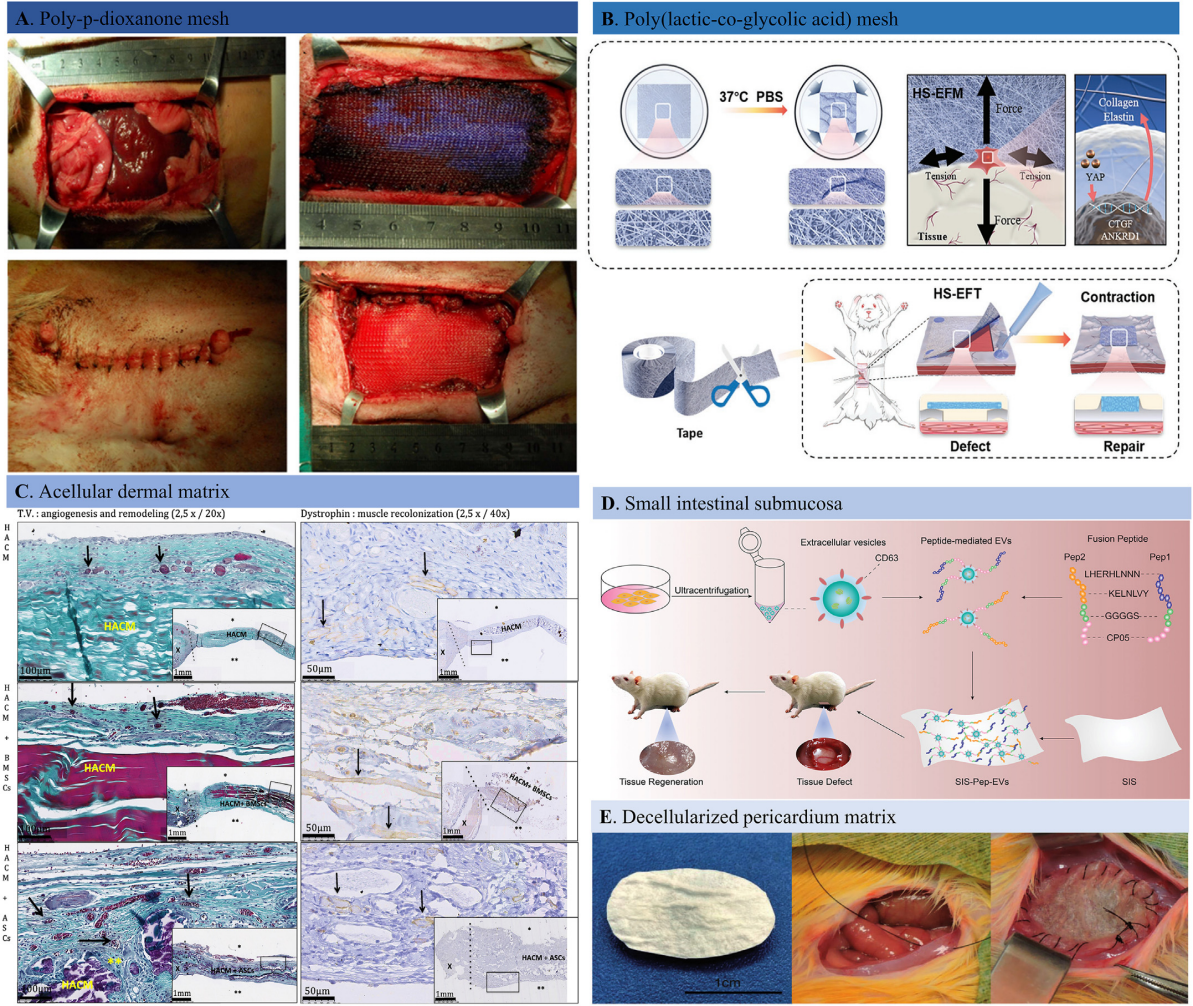
Adsorbable hernia meshes. A. Surgical procedure performed for large-size abdominal wall defect reconstruction with a poly-*p*-dioxanone mesh [171]. B. Schematic diagram of a heat-shrinkable electrospun fibrous tape for reconstructing soft tissue both structurally and functionally in a rat abdominal wall hernia [73]. C. Representative images of Masson's trichrome and dystrophin staining images of explanted grafts after 30 days of implantation. The human acellular collagen matrix group showed no adhesion with or without MSCs. There were different angiogenesis and musculogenesis between groups regarding the matrix loading with different cells [115]. D. Schematic illustration of the small intestinal submucosa (SIS) membrane that modified with fusion peptide-mediated extracellular vesicles which can promote cell migration and spreading, achieving a more successful abdominal wall tissue regeneration [103]. E. Representative pictures of the decellularized and crosslinked bovine pericardial implants, the full-thickness abdominal wall defects induced by sectioning ventral muscular tissue, and the defect reconstructing with implant [157].

Fully absorbable meshes can be absorbed or discharged from the body after serving their reconstructive function, avoiding the unnecessary foreign body reaction that is common with non-absorbable hernia meshes. The absorbable meshes were mainly made of biodegradable polymers or polymer composites, such as polylactic acid (PLA), polyglycolic acid (PGA), Poly (lactide-*co*-glycolide) (PLGA), poly-*p*-dioxanone (PDO), and poly-4-hydroxybutyrate (P4HB) [[76], [80], [169], [170]]. The Vicryl® by Johnson & Johnson, and the Dexon® by Covidien are the PLGA-based hernia meshes that reportedly to degrade within 12 weeks after implantation. TIGR® is another absorbable hernia mesh marketed by Novus Scientific Company, which has a longer degradation period (three years). The reported synthetic absorbable hernia meshes were summarized in Table 3. In general, excellent healing performance was observed during long-term hernia repair using these absorbable materials without severe mesh-related complications such as persistent inflammation and chronic pain [[81], [84], [85], [86]]. For example, the effectiveness and safety of biodegradable PDO mesh for reconstructing large-size abdominal wall hernia have been reported (Fig. 4A) [171]. In vivo animal studies showed that the PDO mesh group exhibited less foreign body reaction, peritoneal adhesion, and mesh infection with higher reconstructive efficacy. In another similar study, a composite mesh made of co-knitted biodegradable P4HB and PGA fibers chemically modified by sodium hyaluronate (HA), carboxymethylcellulose (CMC), and polyethylene glycol (PEG)-based hydrogel on the surface was applied for ventral hernia repair [172]. The mild inflammation, fibrosis, and reduced foreign body reaction at the wound sites indicated its biocompatibility and safety for long-term hernia reconstruction. There are also reported absorbable composite hernia meshes that are made of two or more degradable components, designed with the aim of reducing foreign body reaction, bacterial infection and mesh contraction. In one of our latest studies, we designed a fibrous tape made by PLGA polymer that can tighten the loose abdominal wall fascia under body temperature (Fig. 4B). The in vivo results confirmed its reconstructive efficacy by promoting tissue regeneration before its complete degradation with good biocompatibility and mechanical strength [73]. Despite the fact that the absorbable hernia meshes have conquered most of the problematic complications of permanent meshes, the poor mechanical performance and quick degradation rate limited their long-term reconstructive efficiency. Besides, the degradation products may also trigger immune response which is a major drawback, especially when placed in pre-contaminated hernia situations.

#### 3.2.2 Biological tissue grafts

The concern of chronic pain, mesh erosion, surgical infection, and granulation with commercial prosthetic meshes has pushed biomedical engineers and surgeons to explore alternative options to achieve tension-free hernia fixation in a single-stage surgery or even in highly contaminated situations. Biological grafts, as alternatives to synthetic commercial meshes, were introduced to repair a hernia, holding a promise of a durable repair with fewer post-operative complications than commercial synthetic meshes [[173], [174], [175], [176], [177], [178], [179]]. Biological grafts used for hernia repair are produced by tissue decellularization, including human (allogenic) [[110], [111], [112]] and animal (xenogenic) dermis (Fig. 4C) [[128], [129], [130], [131]], porcine small intestinal submucosa (Fig. 4D) [[87], [88], [89], [102]] and bovine pericardium (Fig. 4E) [[154], [155], [156]] or other organs through various decellularization approaches (physical, chemical, and enzymatic) to prevent infections as well as foreign body reactions. There are commercially available biological tissue grafts for abdominal wall hernia repair, such as Permacol™, Surgisis®, SurgiMend™, XenMatrix™, FlexHD™, Veritas®, AlloMax™, Periguard®, and Alloderm®. The extracellular collagen matrices can positively incorporate into surrounding tissues by fast tissue ingrowth and neovascularization to promote a strong, durable integration of the patient's own tissues. During the incorporation process, the biological grafts gradually degrade and synchronously remodel into newly regenerated tissue to hold the abdominal cavity contents. Ghetti et al. evaluated the post-operative morphological response of human decellularized matrices in hernia patients after one-year post-transplantation [113]. The observed results were that the implant site was characterized by regenerative cellular recruitment, neo-vascularization, reduced inflammatory response, and a well-organized collagen matrix. Hoganson and colleagues reported a newly developed acellular porcine dermis matrix that can successfully retain ECM components such as proteins, glycosaminoglycans and cytokines (VEGF, TGF- β) which will contribute to the rapid graft integration and wound healing [129]. Co-cultured fibroblasts displayed typical spindle-like morphology and excellent viability after one and two weeks. The abundant bioactive components and appropriate mechanical strength promised the decellularized dermis material's great potential for clinical applications. In addition, biological meshes have been accepted to resist infection when implanted, and removal is not required when infection happens [177,180]. Given that, biological meshes were especially useful for body trunk defects and complex defects as there are high risks of infection. In a retrospective cohort study, 64 patients who received complex incisional hernia reinforcement surgery with decellularized urinary bladder matrix were followed for 12–70 months [146]. Despite the complexity and adverse clinical factors involved in three cases, the overall successful resolution of the abdominal hernia within three years with a low rate of complications shed light on a promising strategy for complex abdominal wall hernia repair.

The collagen-rich scaffolds sometimes underwent crosslinking with other chemicals to render stability against collagenase activity and slow down or stop the degradation of the donor collagen [181]. The variety of crosslinking agents and their quantities is critical for increased durability and mechanical strength. On the other hand, however, crosslinking may hinder cell ingrowth due to the reduced scaffold pore size, leading to bacterial infection and fibrotic granulation development. Decellularized and crosslinked bovine pericardial tissue of different degrees for reconstructing a rat full-thickness abdominal wall defect was proposed (Fig. 4C) [157]. They detected the microstructural changes of the implants and explants using second harmonic generation microscopy and found minor changes in collagen fiber organization in crosslinked implants and decreased peritoneal surface erosion, confirming its delayed in vivo degradation. However, fibroblastic cell infiltration, neovascularization, and tissue integration were impeded after crosslinking, hampering host tissue regeneration.

For years, both the clinical data and laboratorial results have suggested that the biological tissue grafts can be applied in infected abdominal wall defects as well as in noninfected fields. Nevertheless, surgical practice using biological grafts is still insufficient compared to the cumulative procedures of synthetic meshes, making it difficult to evaluate the long-term efficacy as there are no high-quality studies on a large number of patients [182,183]. The existing scarce case reports and clinical trials have reported that using biological tissue grafts played only a temporary bridging role, especially in contaminated circumstances, compromising its clinical promotion potential. In addition, some complications such as seroma formation, hemorrhagic complications, high recurrence rate, and post-operative laxity are the unsolved complications that limit its use below the arcuate line [184]. Patrick et al. investigated long-term outcomes of patients who received abdominal wall reconstruction surgery with an acellular dermal matrix in 36 months’ follow-up using the hernia recurrence as the post-surgery evaluation index [114]. The results showed that cumulative hernia recurrence rates reached 11.5% after three years and 14.6% at five years. Moreover, biological tissue grafts have very high costs, which precludes their application in routine abdominal wall reconstruction [185]. Given the unsatisfactory outcomes and high cost, the biological tissue grafts are far from becoming a definitive clinical method of hernia repair.

Taking the advantages and disadvantages of both non-absorbable and absorbable meshes into account, a new generation of composite meshes that are called partially degradable meshes have emerged by combing the non-absorbable and absorbable meshes with the hope of creating orientationally dependent prostheses.

## *4* Engineered prosthetic meshes

Single material or structure based meshes have their own advantages and disadvantages. To give full play to their respective advantages and achieve ideal repair results, it can be expected to engineer a composite hernia mesh that will improve the performance of individual mesh by either combining two or more components or just surface coating with different materials. According to the fabricating method, engineered meshes can be divided to composite meshes and surface coated meshes.

### 4.1 Composite meshes

**Fig. 5:**
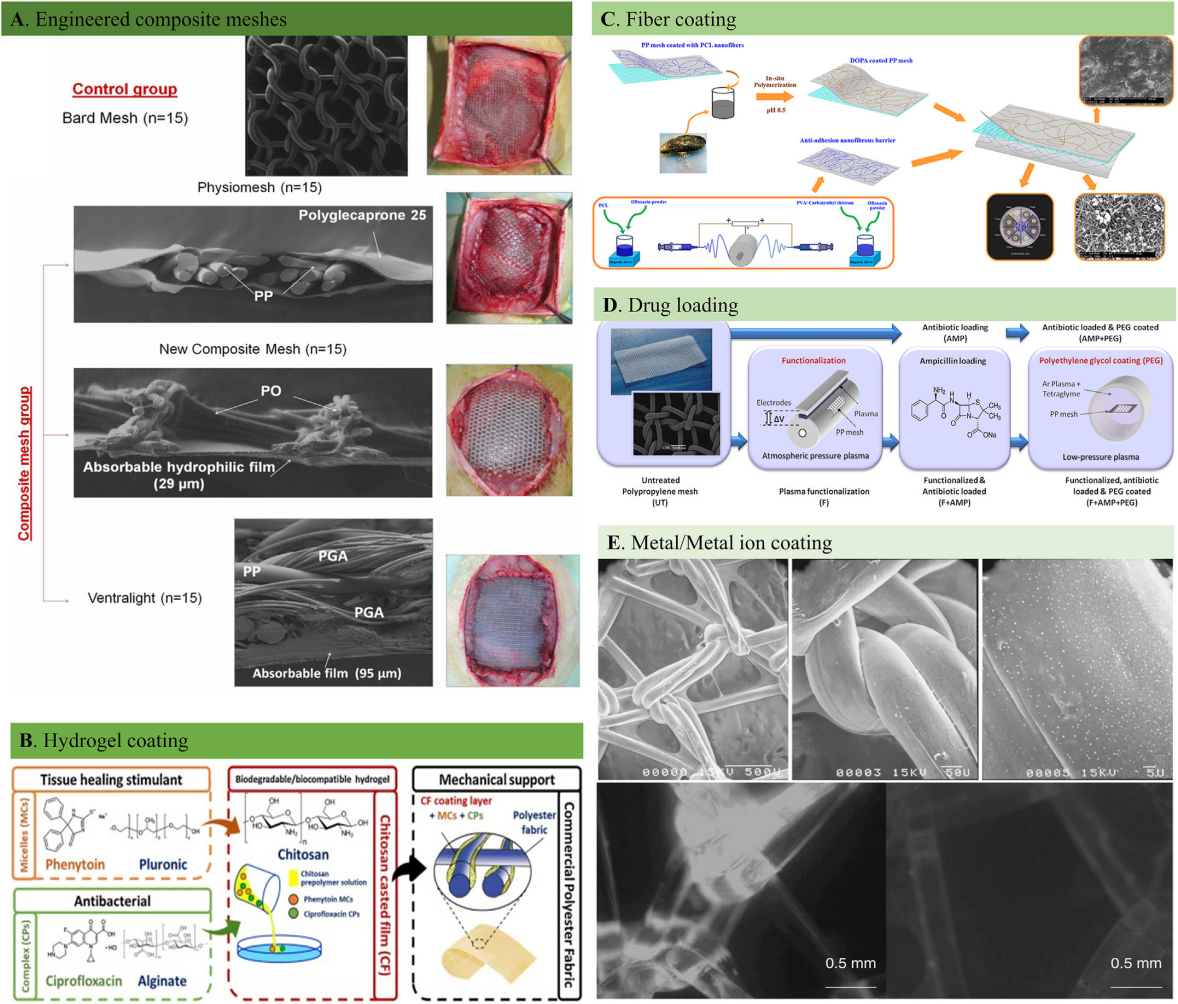
Engineered composite hernia meshes and surface modified hernia meshes. A. Scanning electron microscopy images and macroscopic appearance of the of the commercial and composite meshes [186]. B. The schematic diaphragm of the design, development and evaluation of a new multifunctional prosthetic mesh for abdominal wall defect treatment. The developed hernia mesh is composed of a synthetic commercial polyester fabric coated with a natural biodegradable, biocompatible and antimicrobial layer of chitosan [187]. C. Schematic description of a modified PP mesh coated with two different sides. The backside: PCL nanofiber modified with l-DOPA with adhesive properties; the front side: CECS/PVA and PCL nanofibers with different amounts of ofloxacin as an anti-adhesion barrier [188]. D. Experimental design of an enhanced hernia PP mesh through antibiotic loading [189]. E. Electron scanning microscopic images of silver-coated PP mesh and in vitro effect of presence/absence of silver on PP implants on biofilm formation by *E. coli* [190].

Despite the breakthroughs made within the non-absorbable with higher mechanical strength and absorbable hernia meshes with improved biocompatibility and degradability, several mesh-related complications such as mesh shrinkage, chronic inflammation and unmatched mechanical properties with local tissues still exit generally. In this situation, engineered meshes have been developed by making the non-absorbable meshes as the basis and combining one or more synthetic or natural components such as ePTFE, polyvinylidene fluoride (PVDF), PLA, PGA (Fig. 5A) and collagen into their content. Composite meshes manifest the mechanical handling properties of traditional non-absorbable repair meshes such as PP and PET, while alleviating the overall mesh-related complications by utilizing of absorbable mesh structures. For example, Medtronic Company compounded PET mesh with absorbable collagen membrane, and launched Symbotex™ composite mesh with good tissue ingrowth and anti-adhesion properties. Proceed® is another composite mesh launched by Johnson & Johnson company that is composed of PP and oxidized regenerated cellulose (ORC). PP substrate provides mechanical support and allows tissue ingrowth, while the 10.13039/100013350ORC reduces the adhesion between the mesh and host tissues. The classification of the engineered composite meshes is displayed in Table 4.

**Table 4:** Engineered composite meshes.

	Component	Brand	Advantages	Disadvantages	References
Composite Meshes	PP ​+ ​ePTFE	Composix	Less adhesion formation; Good tissue compliancy	Mesh contarction	[191,192]
	PP ​+ ​Polyglactin	Vicryl	Inflammation; Fibrosis	Poor mechanical strength	[193]
	PP ​+ ​Collagen	Parietex	Low incidence of adhesions; Elevated tissue incorporation	High degree of mesh retraction	[194]
	PP ​+ ​PGA	Dexon	Rapid tissue maturation;	Increased tissue stiffness	[195,186,196]
	PP ​+ ​PVDF	Dynamesh-IPOM	A lower incidence of recurrence, seroma and haematoma formation	Mesh-penetrating adhesions; Bowel obstruction	[197,198]
	PP ​+ ​PLA	Parietene Progrip	Reduced cell-mediated immune response	Inadequate tensile and bursting strength	[199,200]

The main improvement of these engineered composite meshes builds upon the fact that these can provide sufficient mechanical strength while the inertness, hydrophobicity, or foreignness could be adjusted to minimize unwanted complications to the least degree. Moreno et al. compared the post-surgery complication of three different commercial hernia meshes by implanting them in the rabbit umbilical hernia model [195]. After evaluating the tissue ingrowth and inflammatory response at the implant sites, the team found Ventralex ST hernia mesh which is composed of PP and PGA, and Ptx that is a hernia mesh made by PET and collagen composition, showed satisfying mesothelialization with a minimum adhesion formation. On the contrary, the Proceed mesh, made of multiple layers that contain non-absorbable polypropylene and absorbable oxidized regenerated cellulose components, triggered comparable inflammatory cell response at different time points. Novitsky et al. studied the post-implant adhesion formation and new tissue regeneration difference between the PP, ePTFE, PP ​+ ​ePTFE composite mesh, and ORC ​+ ​PP composite meshes in a rabbit abdominal wall hernia model [191]. After one year of implantation, the overall mesh efficacy was evaluated regarding the adhesion score, mesh shrinkage, and the strength of the implant site. The results confirmed that the PP caused as much as 40% of tissue adhesion while the ePTFE triggered serious mesh shrinkage despite its highest mesh compliancy. On the other hand, the composite meshes displayed the best hernia repair performance. Taken together, the study demonstrated that the long-term effects of composite prostheses are superior to the single meshes regarding minimizing long-term postoperative morbidity.

Literature results over decades proved that the composite meshes indeed outperformed in certain aspects during the hernia reconstruction. However, this composite mesh is usually made of thick materials with large area density and poor abdominal wall compliance after implantation. In addition, as a permanent foreign body, the composite meshes are prone to cause chronic inflammation and other side effects, limiting their application range in the clinic.

### 4.2 Surface coated meshes

Despite numerous improvements, the complications encountered with the biological tissue grafts and composite hernia meshes, such as fistula formation, poor tissue ingrowth, foreign body reaction, and mesh-induced infection, brought the new mesh engineering concept through surface modification. Patient-friendly and functional prosthetic meshes can be designed by modifying various functional agents onto the surface of non-absorbable commercial meshes by biological, chemical, and physical treatments. Metallic ions or metal-based nanoparticles [201,202], hydrogels [[203], [204], [205]], fibrous membranes [206,188], drugs or bioactive agents [207], and cells or tissues [208,209] are the common coating options for the surface modification process. The most commonly reported surface modification approaches were displayed in Table 5. The main focus of the overall engineering processes is to functionalize the meshes with anti-adhesion [210,211], anti-inflammation [212,213], antibacterial [[189], [214], [215]] and regenerative properties [216,217] without sacrificing their mechanical strength.

**Table 5:** Surface coating approaches of engineered meshes.

Classification	Component	Advantages	Disadvantages	References
Metals	Ti	Improved hydrophilicity, biocompatibility	Complex coating procedure	[[218], [219], [220], [221], [222]]
	Ag	Anti-infection and biofilm inhibition properties	Delayed healing; Adhesion formation	[215,190,[223], [224], [225], [226]]
	Zn	Anti-bacterial properties	Adhesion formation	[214]
Synthetic Hydrogels	GelMA PEG PVA Synthetic HA derivatives Poly-glycidyl methacrylate/human serum albumin (PGMA/HSA) PNIPAAm ORC	Improved cellular response; Post-operative adhesion prevention; Anti-infection effects; Enhanced tissue integration; Incresed biocompatibility; Partial mesh biodegradability	Inflammation and fibrosis; Poor durability; Toxic degradation products; Complex coating process; Harsh synthesis process	[203,210,227,[228], [229], [230], [231], [232], [233], [234], [235]]
Natural Hydrogels	Collagen Fibroin Chitosan Gelatin β-Glucans Fibrin	Excellent biocompatibility; Improved tissue integration; Rapid tissue maturation	Long-term instability in protective effects; High cost; Poor manageability; Low extraction efficiency	[204,211,236,237,187,[238], [239], [240], [241], [242], [243], [244], [245], [246], [247], [248], [249], [250], [251], [252], [253]]
Fibers	PCL PLA PLGA	Anti-adhesion barrier; Anti-infection properties; Drug loading capacity	Fiber droppage in vivo; Toxic degradation products; Inertness-induced inflammation	[206,188,217,[254], [255], [256], [257], [258], [259]] [260,199,200,261] [262]
Chemical Functionalizing	Drugs Polymers	Anti-bacterial function; Anti-adhesive capability; Anti-inflammation	Low drug loading capacity; Drug burst release; Instability of drugs; Limited drug carrying pattern	[207,189,[263], [264], [265]] [213,[266], [267], [268]]
Bioactive Components	ECM Fibroblasts Stem cells Stromal cells	Anti-inflammation; Improved immune cell response; Enhanced tissue ingrowth; Biocompatibility; Rapid Collagen deposition	Post-implant infection; Poor mechanical strength; Higher fabrication demand; Elongated preparing process Poor practicability	[212,269,270] [209,271,272] [208,[273], [274], [275], [276], [277], [278], [279]] [280]

As the repair meshes are directly in contact with intraperitoneal contents, it's inevitable to induce abdominal adhesions if the hernia site is left untreated. In this context, surface modification for inhibiting visceral adhesion is particularly important. In most situations, a functional hydrogel can be coated onto the hernia mesh to prevent the visceral adhesions (Fig. 5B). For instance, Hu et al. investigated the anti-adhesion function of a newly developed hydrogel-PP mesh composite both in vitro and in vivo [236]. The in-situ hydrogel coating layer is made of dopamine and carboxymethyl chitosan in crosslinking agent-free way. Apart from the in vitro biocompatibility, the gel/mesh composite effectively alleviated the local inflammation and collagen deposition around the mesh without interrupting the mesh-abdominal wall integration.

The post-surgery infection is a major issue in hernia repair, inhibiting wound healing and requiring additional surgeries. Direct drug loading onto the hernia meshes is one of the practical approaches to achieving anti-bacterial functionalities (Fig. 5D). In a related study reported by Labay and the colleagues, the PP meshes were loaded with ampicillin after exposing to plasma treatment on the mesh surface [189]. The two-step plasma treatment changed the surface bounding sites chemically and morphologically and higher drug loading and better cellular adhesion and migration activities. In vitro antibacterial assay further confirmed the drug-loaded mesh's excellent anti-bacterial activity demonstrating its good clinical potential. Despite the considerable anti-infection efficacy of drug-loaded hybrid meshes, the unsolved low-safety and high-cost problems pushed scientists to develop alternatives for anti-bacterial hernia repair meshes. Based on the bio-functional properties of metal ions or metallic oxides, some solutions were proposed to improve the anti-infection stability of the engineered hernia meshes. In one such study, Yurtkap et al. examined the antibacterial potential of the zinc ion integrated PP mesh in the in vivo studies [214]. A significantly reduced number of CFU was observed compared with the node mesh when placed in a contaminated environment after three months of follow-up.

Foreign body reaction is the first immunological response to an inert material when inserted into the body with varying degrees of intensity and chronicity based on the nature of the implant. The body's self-protecting strategy is to promptly remove the danger and trigger the upcoming repair procedures. Given that, designing immunologically friendly hernia repair meshes became the main trend to enhance the morbidity of the abdominal hernia. Much research on mesh modifications has been explored in this given context, using specific implant architectures and various active coatings. Extracellular matrix components, biocompatible hydrogels (Fig. 5A), fibrous membranes (Fig. 5C), and functional agents (Fig. 5E) were among the earliest approaches to alter in vitro cytocompatibility and in vivo histocompatibility to promote hernia repair. Qiao et al. have employed a hydrogel layer composed of chondroitin sulfate and gelatin crosslinked by tannic acid on the PP mesh with the aim of providing a biomimetic surface [237]. Interestingly, the fabricated gel-mesh composite achieved coordination between the anti-inflammatory properties of the coated hydrogel and the good mechanical strength of the PP mesh. The authors concluded that the facile approach to develop an immunologically friendly hernia mesh induced a limited collagen deposition around the implants, while increasing vascularization and promote the regeneration of native tissue compared with the new PP mesh. Non-specific protein adsorption triggering immune cell recruitment around the implanted mesh also leads to foreign body reactions impeding implant integration and tissue regeneration. Thus, engineering PP meshes to minimize non-specific protein adsorption may be an efficacious solution for repressing immune cell recruitment and activation, eventually contributing to in vivo tissue regeneration. Wang's group established dopamine-mediated zwitterionic poly (sulfobetaine methacrylate) (PSBMA) coatings to prevent protein adsorption onto the hernia meshes by two strategies (sequential deposition and co-deposition) and confirmed less macrophage adhesion and proinflammatory cytokine release without sacrificing the mechanical properties of PP mesh [281].

Direct regulation of immune cell fate through surface coating is another effective way to reduce mesh-related inflammation. For example, regulating the ratio of macrophage subtypes (M1/M2) can effectively attenuate the post-implantation immune response of mesh materials. In this regard, macrophage polarization effects of an extracellular matrix coated PP mesh were investigated in vivo and found that the ECM coating significantly reduced the M1 (pro-inflammatory phenotype) response and foreign body giant cell aggregation around the implanted mesh spatially and temporally [269]. To command post-implantation foreign body reactions, biologically derived components are subsequently investigated by various research groups hoping that cells should enhance the bio-compatibility and reduce foreign body reactions (Fig. 5E). Several cell types were selected to cover the hernia meshes, including fibroblasts [209,271], skeletal muscle cells [208], and stem cells [[273], [274], [275], [276], [277], [278]]. All these research results demonstrated beneficial outcomes considering tissue response and regeneration of the hernia meshes. Adipose-derived stem cells (ADSCs) can self-renewal, pluripotency, and differentiation, therefore displaying multiple features for tissue engineering material designing. Preliminary experiments published by Zhao et al. clearly highlighted the benefits of ADSC coating on the PP mesh in rats [279]. The team found that after implanting normal and ADSCs coated PP mesh under the abdominal muscular layers, the number of immune cells showed a significant difference between the two groups indicating its hernia repair potential in a tissue-friendly way. Apart from the above strategies to fight mesh-related complications, numerous scholars have also investigated some engineered composite meshes over the last decade. Both degradable and non-degradable components were combined with commercial prosthetic hernia meshes to improve tissue integration and enhance the chance of adequate hernia repair [195,282,186].

As one design may exhibit clear advantages compared with the first generation of hernia repair meshes, it is difficult to pinpoint a single mesh that integrates all of the properties of an ideal mesh. The endless efforts of surface coating or combining degradable components have partly solved the mesh-related complications. However, instability of surface coating and complex procedures limit its broad application in clinical. Besides, internal organ adhesion, bacterial biofilm development, mesh device failure, mesh encapsulation, and insufficient mechanical support are sill the main drawbacks that engineered meshes cannot rid of.

The overall goal of hernia repair is to reconstruct the abdominal wall integrity with well-matched mechanical strength provided by hernia plastics. In recent years, some scholars have proposed that ideal hernia repair meshes should generally meet the following performance requirements: 1. No toxic and side effects; 2. Sufficient mechanical strength and stable physical and chemical properties; 3. Easy to operate without displacement; 4. Anti-adhesion and anti-infection properties; 5. Cost effectiveness. Although the perfect mechanical strength was provided by the commercial hernia meshes of different types during the past decades, there has not an ideal option to cover all the requirements up. Due to the overwhelming incidence rate of the hernia and mesh-related post-surgery complications, there is still urgent yet unmet demand for hernia repair device development. To this end, as a rising star in healthcare field, tissue engineering and regenerative medicine has brought a promising future for successful hernia repair. Hybrid tissue engineering materials allow for the improvement of the biological properties of materials and have been successfully used for implantology in hernioplasty. In the following section we review the smart materials that designed for hernia repair systematically.

## 5 Smart engineered materials for abdominal hernia

A range of biomaterials has been developed as science and technology sought to meet the huge clinical demand to repair and regenerate injured tissues suffered from trauma or disease in the human body [283]. Tissue engineering smart scaffolds with various functionalities have provided insights for treating abdominal wall hernias [284]. Tissue engineering aims to recover the function of injured, lost, or aged tissues by combining target cells, drugs, and engineered scaffolds [285,286]. Different types of smart engineering materials are used in tissue regeneration, including but not limited to hydrogels [[287], [288], [289]], fibrous membranes [[290], [291], [292], [293], [294], [295]], and 3D printing scaffolds [296,297]. In this section, we mainly focus on the common smart hernia repair scaffolds that have been reported for hernia repair in recent years.

### 5.1 Hydrogels

Hydrogels are the 3D network of polymeric structures that can absorb a large amount of liquid with controllable network density, mechanical properties, and degradation rate, which enable researchers easily modify them to widen their application range [298]. Hydrogels can be made from natural or synthetic polymers through various approaches, which can be rendered with myriad functions—for example, pH-responsiveness, thermos-responsiveness, photo-responsiveness, electro-conduciveness, and self-healing properties. The synthetic and natural hydrogels are structurally akin to tissue ECM that can be therapeutic cell delivery vehicles and in vitro tissue models. However, despite these exciting structural and functional developments, hydrogels have also been subject to criticism. Fast degradation rate, weak mechanical strength, homogeneous structure, and static physicochemical properties are the main challenges keeping hydrogels from becoming the forefront of cutting-edge biomaterial research. Hernia repair in nature is mechanically strengthening the weak and loose parts of the abdominal wall with relatively strong meshes. In this situation, hydrogels are obviously not the first option. However, the chemical modifiability and adjustability made it possible for researchers to make tougher hydrogels with excellent anti-adhesive properties [299,227]. According to the source, the hydrogels used in hernia repair can be classified into natural and synthetic hydrogel scaffolds. The specific classification of hydrogel hernia meshes were displayed in Table 6.

**Table 6:** Hydrogel hernia scaffolds.

Classification	Component	Functions	References
Natural Hydrogel Scaffolds	Collagen	Matrix deposition, angiogenesis, adipogenesis and skeletal muscle regeneration; Reduced postoperative adhesion, induced tissue re-mesothelialization	[284,300,301]
	Chitosan	Quick cellular response, rapid ECM deposition, and improved neovascularization	[302]
	Gelatin	Mild postoperative adhesion, sufficient neovascularization, induced collagen deposition and better collagen organization	[303]
	ECM Matrix	Anti-inflammatory response; Elevated myogenic differentiation; Reduced scaffold encapsulation; Increased wall thickness and enhanced cellular infiltration	[304,305,306]
	Cellulose	Effective prosthesis integration, induced tissue remodeling	[307]
	Fibrin	Reduced hernia recurrence, increased fibrosis	[308]
Synthetic Hydrogel Scaffolds	HA derivatives	Ameliorated postoperative peritoneal adhesion; Effective defect sealing	[309,310]
	GelMA	Anti-inflammation; Rapid collagen deposition	[311]
	PVA	Anti-Deformation, anti-adhesion, and pro-healing characteristics	[312]
	Resilin	High strength and super-toughness, outstanding biocompatibility, and reduced tissue adhesion	[313]

#### 5.1.1 Natural hydrogels

**Fig. 6:**
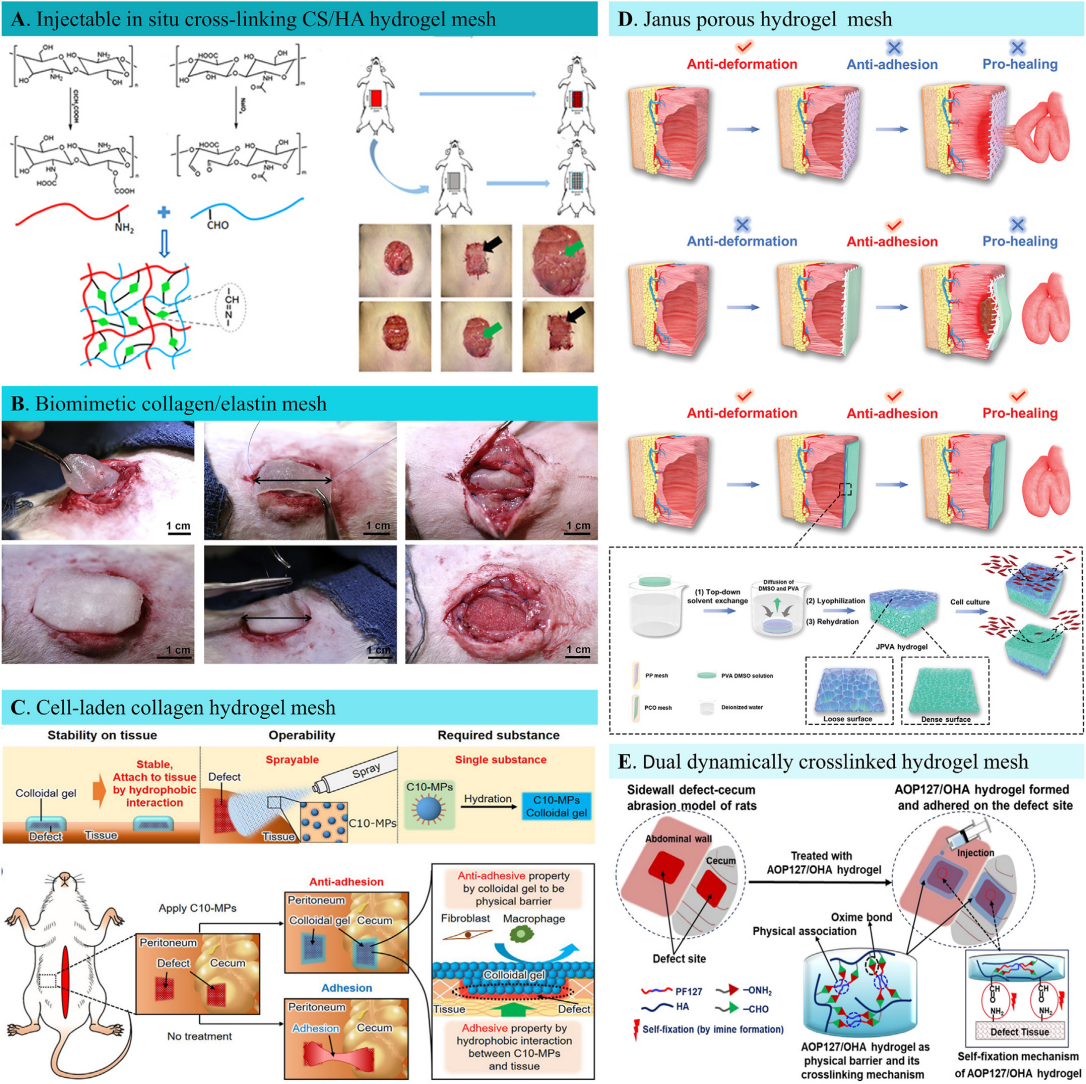
Hydrogel based hernia meshes. A. Schematic illustration of preparation of CS/HA hydrogel via Schiff's base reaction for abdominal wall hernia treatment [302]. B. Rat ventral hernia repair surgery with the implantation of a type I collagen/elastin crosslinked blend (CollE) hernia mesh [284]. C. Schematic characterization of a microparticle based, sprayable adhesion prevention hernia mesh composed of decyl group modified Alaska pollock gelatin (C10-ApGltn). Characteristics of the new colloidal gel barrier to prevent postoperative adhesion [300]. D. Schematic illustration of structure and abdominal wall defect repair properties for conventional hernia meshes, and a brand-new Janus porous poly (vinyl alcohol) hydrogel patch, which is prepared through top-down solvent exchange, lyophilization, and rehydration processes [312]. E. Schematic demonstration of fabricating a dual dynamically crosslinked hydrogel to serve as a physical postoperative anti-adhesion barrier generated by alkoxyamine-terminated Pluronic F127 and oxidized hyaluronic acid [309].

Collagen, fibrin, elastin, alginate, chitosan, hyaluronic acid are the examples of natural hydrogels used in tissue engineering. Natural hydrogels can address the clinical need for using the biomimicry principles to better simulate the native tissue's architectural structure, biochemical composition, and mechanical strength. Deng et al. designed a chitosan-hyaluronic acid (CS/HA) hydrogel through Schiff's base reaction with suitable mechanical and biological properties for rat large abdominal wall defect reconstruction (Fig. 6A) [302]. The obtained hydrogel is softer with a Young's modulus of 34 ​Pa, but it is robust enough for abdominal wall regenerative application. The hydrogel's porous structure promoted cellular ingrowth by facilitating nutrition infiltration. During the slow degradation process, CS/HA hydrogel induced native tissue regeneration, which is 2.5 folds thicker than the control group. This can be attributed to the abundant regenerative cell (fibroblasts and endothelial cells) repopulation within the hydrogel. Rich collagen components and a newly formed vascular system are also detected in the hydrogel, confirming its successful reconstructive capacity. In another study by Minardi et al., a new off-the-shelf biomimetic mesh was constructed by crosslinking the type I collagen and elastin (Fig. 6B) [284]. The obtained CollE-based biomimetic mesh was successfully integrated into the native tissue after six weeks of the implant with fine vascularization and collagen deposition. The mechanical strength of the biomimetic mesh was similar to native abdominal wall tissue, which is supportive for long-term tissue regeneration. Despite the exciting restorative ability of the natural hydrogels with fine histocompatibility, low immune response and little foreign body reaction, the poor mechanical strength has made them be subject to wide criticism. To render better mechanical properties, scholars have explored new chemical strategies (eg. in situ crosslinking, injectable delivery), synthetic designs (eg. pH responsiveness, thermos responsiveness, photo sensitiveness), and fabrication methods (click reactions).

#### 5.1.2 Synthetic hydrogels

With the gradual rise of precise medicine, regenerative medicine has stepped into concise structural and functional regulation of damaged tissues. Synthetic hydrogels, with optimal functionalities, can meet the desired requirements for regenerating target tissues with similar biochemical and structural properties. Synthetic hydrogels are generally obtained by chemical modification of specific bonds, functional groups or responsive sites. The complexity of synthetic hydrogels also affords ECM mimetics a large range of possibilities to explore, in the demand for novel biomaterials for hernia repair. Importantly, in the area of biomaterials research, sensitive hydrogels or smart hydrogels has been gaining greater momentum. Scholars have recently designed various functional synthetic hydrogels for abdominal wall defect reconstruction in recent years, including immuno-regulation [304], angiogenesis [314], neurogenesis [315,305], and anti-adhesion properties [300]. The precise intervention of clinical scenarios in hernia repair with hydrogels has yielded satisfactory results. For example, in one of our recent studies, an anti-inflammatory Chinese traditional drug-loaded gelatin methacryloyl (GelMA) hydrogel achieved local immune microenvironment regulation in a rabbit abdominal wall hernia model [311]. The porous hydrogel inhibited neutrophil and eosinophil recruitment into the wound area, activating the TGF-β/MMPs pathway to promote fascia regeneration. As early immune regulation aided ECM remodeling, spatial and temporal muscle fascia reconstruction was achieved, promising an excellent potential for hernia repair.

One of the most challenging issues regarding hernia implants is their foreignness to surrounding tissue and aptness to cause visceral adhesion. Engineers have proposed some strategies to circumvent these undesirable effects, such as collagen coating polyester or PP scaffolds, providing a smooth visceral side that can act as an anti-adhesive, non-erosive, and antibacterial barrier [303]. Nevertheless, the scaffolds resulted in poor wound healing due to their cellular unfriendly microstructures and easy deformation within wet environments. It is always the unsolved challenges that encourage scientists to come up with innovative strategies. Designing advanced scaffolds for efficient hernia repair is one of such challenges for both engineers and surgeons. Hydrogel-type anti-adhesion barriers possess good injectability, and quick gelation ability in situ by simply mixing two or more components through a syringe. Ito et al. reported a colloidal gel anti-adhesive barrier prepared by Alaska pollock gelatin made tissue adhesive microparticles for intraperitoneal adhesion prevention [300]. The simple colloidal gel formation by hydration proved to be stable in water, and better tissue followability for effective adhesion prevention in vivo (Fig. 6C). However, the preparation process of the hydrogel barrier system is too complicated. Although the system demonstrated excellent anti-adhesive properties, a totally cell-resistant scaffold is not good enough for abdominal defect reconstruction. Moreover, the regenerative capacity should also be taken into account when designing hernia repair materials, as the defect itself always main target for strengthening the abdominal wall. All these demands have pushed scientists to take further step into fabricating multifunctional meshes that can meet both anti-adhesive and pro-regenerative needs in herniaplasty. A more recent cutting-edge and inspiring antiadhesive repair solution reported by Liang et al. [312] was just an attempt to this demand. A biocompatible Janus porous poly (vinyl alcohol) (PVA) hydrogel was developed with a dense, smooth anti-cell adhesion surface and loose ECM-like porous and rough surface (Fig. 6D). The structurally peritoneum bionic hydrogel displayed a meager swelling property which guaranteed its anti-deformation performance and strong mechanical strength. In vitro cell culture results confirmed that the porous top surface facilitated regenerative cell adhesion and proliferation while the dense, smooth bottom surface acted contrarily by inhibiting fibroblast adhesion. After being applied in a rat full-thickness abdominal wall defect model, the Janus hydrogel efficiently inhibited visceral adhesion with an adhesive score of 0, forming a sharp contrast with PP mesh which triggered severe implant-visceral adhesion with the adhesive score being as high as 9.8. In addition, the pro-healing features were evaluated by histological studies, and the attenuated immune cell infiltration, faster collagen deposition, and higher angiogenesis rate within Janus hydrogel indicated better wound healing properties compared with sham and PP mesh groups. The unique anti-adhesive character and excellent tissue regeneration capacity made the Janus structure hydrogel a promising abdominal wall hernia repair platform.

Despite the marvelous achievements made in hydrogel hernia repair scaffolds, including a myriad of chemical and physical strategies for anti-adhesive hernia repairing (Fig. 6E), the fatal weakness of poor mechanical properties and easy degradation has not been solved ideally yet [307]. Under such circumstances, scholars have kept searching for new solutions for better treatment of abdominal wall hernia for decades. Inspired by numerous diseases that benefited from tissue engineering, many other smart hernia repair materials have come into the scientific community.

### 5.2 Electrospun fibrous meshes

Although the mainstay surgical meshes for hernia repair have changed over decades from synthetic prosthetics, engineered surgical meshes, and biological tissue grafts to smart devices, tension-free abdominal wall reconstruction is the final goal. Mechanical support and tissue ingrowth are the least basic requirements for hernia reconstruction material design. Hydrogels promoted successful tissue integration due to the ECM-like 3D structure and tunable morphology. However, the weak mechanical strength and fast biodegradation have limited its wide application in clinics. Electrospinning, another tissue engineering approach, brought dawn to mechanical performance problems for hernia repair. Electrospinning is a simple and versatile technique to fabricate scaffolds diameters ranging from several nanometers to micrometers that architecturally resemble ECM and consequently contribute to the emerging field of nanotechnology [316].

Biological active components can be incorporated through polymer selection, chemical modification, and parameter adjustment [317]. Electrospinning acts as an efficient smart platform in a wide range of regenerative tissue applications (tendon, cartilage, cardiovascular, neural, skin, and bone regeneration) as the potential solution to current biomaterial (such as hydrogels) limitations. Coaxial electrospinning [318], direct blending [319], emulsion electrospinning [320], and surface modification [321] are the most common two-dimensional or 3D tissue engineering scaffold fabrication methods. Since most electrospinning materials are hydrophobic and lack bioactive sites for cell recognition and adhesion, fiber functionalization is sometimes necessary for electrospun fibers to be applied in regenerative medicine [322,323]. Given the existing challenges of previous reconstruction materials, abdominal wall hernia repair has also begun to seek a targeted solution from electrospun fiber-based tissue engineering approaches [314,324,325]. Using biodegradable synthetic materials, a fibrous hybrid fiber can be electrospun to be implanted at the abdominal wall defect site to provide the needed mechanical strength and regenerate new functional muscle tissues, promising an alternative to meet current clinical needs [[326], [327], [328]]. Several electrospinning methods have been developed over the past decades to fabricate functional electrospun fibrous membranes for hernia repair. The mostly reported approaches were summarized in Table 7.

**Table 7:** Electrospun fibrous hernia meshes.

Classification	Components	Functions	References
Simple electrospun hernia meshes	PCL	Sufficient biomechanical reinforcement; Enhanced suture retention and tensile strengths	[329,330]
	PLGA	Well-matched mechanical strength, enhanced collagen deposition	[73]
Composite electrospun hernia meshes	PLCL ​+ ​fibrinogen	Moderate mechanical strength; Excellent biocompatibility; Rapid muscle reconstruction	[282,323,325]
	PCL ​+ ​silk fibroin	better cell proliferation microenvironment, increased angiogenesis, less-intensive adhesion formation	[331]
	PLA ​+ ​Collagen	Enhanced cell adhesion and proliferation in vitro; Improved myogenesis and αSMA formation in vivo	[332]
	PCL ​+ ​Upy	Good tissue compliance	[326,328]
	PCLMA ​+ ​GelMA	Tunable mechanical properties, high biocompatibility, less inflammatory response, good biodegradation, and collagen deposition	[321]
	PEUU ​+ ​ECM gel	Proper mechanical strength, extensive cellular infiltration, well tissue integrative behavior	[322,306]
Carrier fiber meshes	Antibacterial agent	Controlled drug release; Inhibited bacterial growth; Reduced immune cell infiltration; Suppressed inflammatory reaction	[327,333,334,335]
	Antiinflammation agent	Local inflammatory regulation; Improved collagen deposition and neovascularization; Attenuated visceral adhesion formation	[318,320,77]
	Cells	Enhanced tissue regeneration, rapid vascularization; good biocompatibility and anti-inflammatory performance	[260,336]
Multi-layer fibrous hernia meshes	PCL ​+ ​graphene oxide ​+ ​chitosan	excellent mechanical strength and biocompatibility; excellent anti-hernia effects; less adhesion formation and more collagen deposition	[319]

#### 5.2.1 Simple electrospun hernia meshes

Tunable fiber morphology, membrane geometry, mechanical property, and in vivo degradability have made electrospinning fibrous membranes popular target for hernia tissue engineering. Compared with the non-absorbable hernia meshes that rely on the fibrotic tissue that composed of disorganized collagen with poor mechanical strength and high recurrence rate, the electrospun fibrous membranes that mimics the natural ECM with porous, nonwoven microstructures. The lightweight substrates are cost-effective, easy to fabricate with great translational potential. Ebersole et al. fabricated PCL electrospun fibrous hernia scaffold with an appropriate mechanical strength [166]. The suture retention strength was ​≥ ​20 ​N, tear resistance ​≥ ​20 ​N, and tensile strength ​≥ ​50 ​N ​N/cm which are critical for successful abdominal wall reconstruction. As the fabricating parameters are essential for electrospun fiber mechanochemical properties, the team compared different polymer concentration and flow rate and verified that higher concentration with lower flow rate can produce ideal mechanical performance. In general, compared with hydrogels, the mechanical properties of the hernia site were guaranteed, but the inertness of the polymer prevented cell adhesion, growth and consequently the tissue integration when applied in vivo. Given that, the fibrous fistula formation, poor tissue ingrowth and wound healing is inevitable for simple electrospun fibrous meshes. To overcome the drawbacks and render the electrospun fibrous meshes with different functionalities, composite solutions were developed by researchers.

#### 5.2.2 Composite electrospun mesh fabrication

**Fig. 7:**
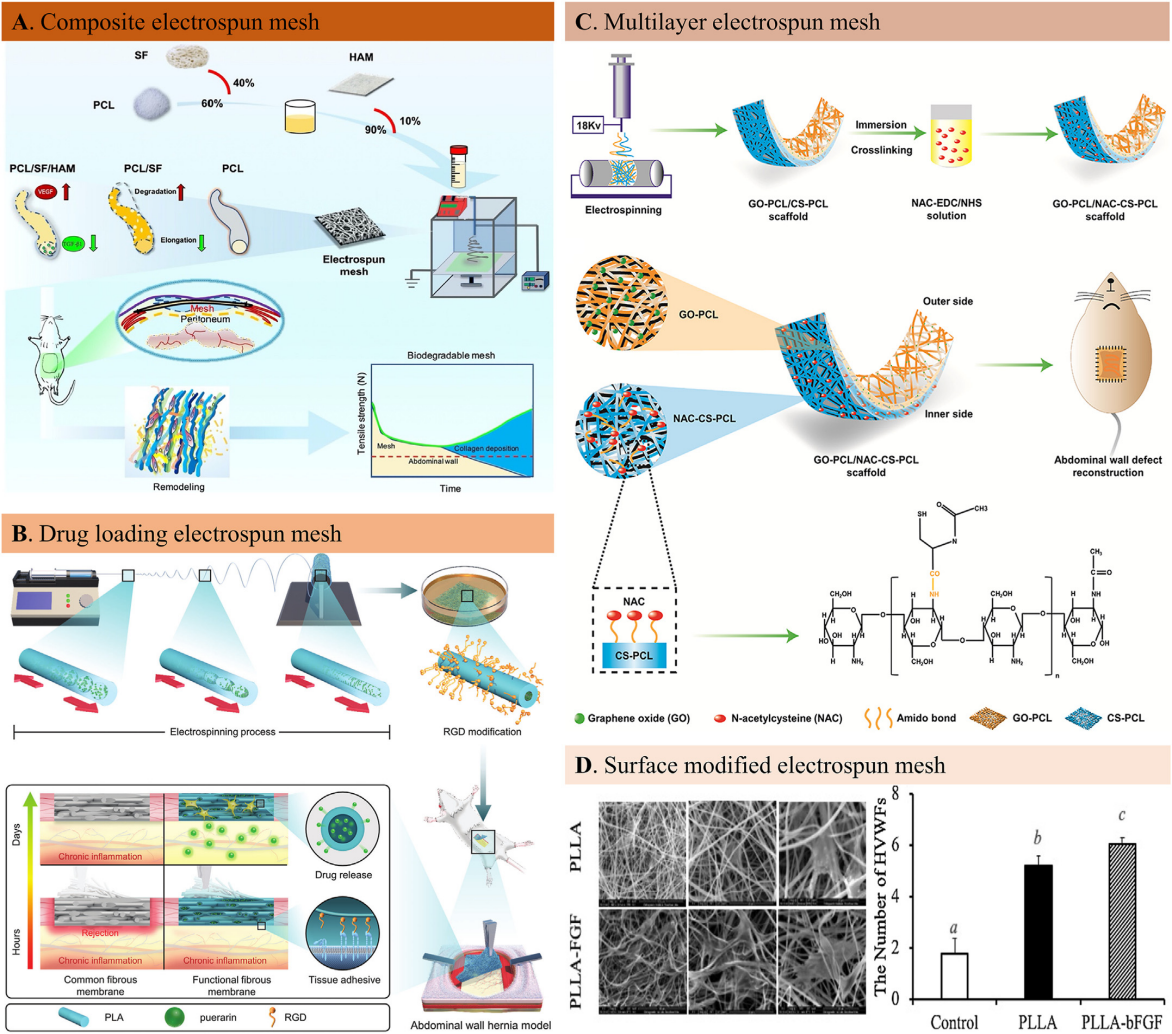
Electrospun fiber-based hernia meshes. A. Schematic illustration of the fabrication of a functional electrospun mesh by combining PCL with silk fibroin (SF) and decellularized human amniotic membrane (HAM) for full-thickness defect repair [331]. B. Schematic illustration of a functional “outer inner” medicated fibrous membrane preparation and the regulated early exogenous and long-term endogenous inflammation process in a rat abdominal wall hernia model. A traditional Chinese medicine was loaded onto the fiber by micro-sol electrospinning technique while a functional peptide was modified on the fiber surface [320]. C. An illustration of the design and fabrication process of a double-layer structured nanofiber membrane made by PCL, graphene oxide and chitosan with excellent mechanical strength and biocompatibility [319]. D. The electron scanning microscopic images of human vaginal fibroblast proliferation on the bFGF-modified PLLA fibers to promote hernia repair and the cell proliferation assay by cell count kit study [77].

The hydrophobicity of the inert polymers is the main reason for adhesion formation, inflammatory reaction and re-herniation due to the poor cell affinity and/or slow degradation. In this regard, mixing the inert polymers with hydrophilic, biocompatible and biodegradable components and fabricating a hybrid mesh is a better option. The hydrophilic and biocompatible components such as fibrin, silk fiber and ureidopyrimidinone can not only improve the cellular aptness of the fibrous mesh, but can be made into biomimetic scaffolds while not sacrificing the mechanical strength of the polymer fiber. As such, developing a functional fibrous patch with appropriate mechanical properties has been a clinically relevant solution. In their recent study, Liu et al. reported a hybrid mesh composed of polycaprolactone (PCL) and silk fibroin (SF) for abdominal wall defect repair [333]. The amoxicillin-loaded multi-walled carbon nanotubes were chemically crosslinked to the fibrous scaffolds to inhibit bacterial growth around the implants (Fig. 7A). The improved hydrophilicity rendered the fibrous mesh excellent biocompatibility for cell adhesion, proliferation, and functional secretion. The chemical modification didn't obviously alter the mechanical strength that perfectly matches to biomechanical properties of the native abdominal wall. The in vivo histological results showed that the inflammation reaction characterized by immune cell infiltration and fibrous encapsulating tissue formation was significantly attenuated in the drug-loading composite mesh group. The well-controlled inflammation and infection resulted in a rapid matrix remodeling in the abdominal wall defect area. Another study reported an electrospun composite mesh made of poly (l -lactide-*co*-caprolactone) (PLCL) mixed with fibrinogen. An optimal blending rate was selected to obtain mechano-physical characteristics (mechanical strength, porosity, hydrophilicity) of this hybrid mesh. After being implanted in a canine abdominal wall defect model, the inflammatory response was significantly reduced while increased myofiber formation with a well-tuned degradation rate. Considering the mesh shrinkage that can be caused by blending polymer with biological components (such as collagen, gelatin, chitin, etc.), the team measured the shrinkage rate of the hybrid mesh a slow as 8% which can retain its mechanical strength after implantation in vivo. Blending the natural components in the electrospun fibrous membranes can provide favorable cell-to-cell and cell-matrix interactions for effective wound healing.

#### 5.2.3 Electrospun fiber mesh carriers

Electrospinning is a globally accepted technique for nano-to-microfiber fabrication given its simplicity, cost-effectiveness, and ability to design multiple applications. What's more, the drugs or other components that solve in the electrospinning solvents can simply functionalize the fibrous structures as effective carriers to treat diseases. Drugs, peptides and even cells can be loaded onto the lectropunk fibrous membranes for specific purposes. The long-term and short-term drug release can be designed according to the disease treatment models by tuning the fiber structural and physicochemical properties. Direct blending, surface modification, core-shell structure fabricating by micro-sol technique is the most commonly adopted strategies for designing electrospun carriers.

One of the severe side effects of non-degradable hernia meshes is implant-related surgical site infection and which causes intraabdominal infection, abdominal pain, intestinal obstruction, and even fistula. To reduce the unacceptable complication with electrospun fiber meshes, antibiotics were loaded to repair hernia in infectious context. In such a study conducted by Barrientos, antibiotic agent levofloxacin or irgasan were loaded onto PCL fibrous meshes. The in vitro release studies showed different release behavior of these two kinds of antibiotics due to different drug release mechanisms. The drug release files of electropsun fibers can be different in different fiber material matrices, release medium and the drug component. So, there can be a wide range of loading choices to achieve higher treatment efficacies. For example, some hydrophilic drugs that cannot be solved inorganic reagents can be loaded to hydrogels such as hyaluronic acid, gelatin or collagen before being packaged into polymers by special ways such as emulsification. In this way, a core-shell structure can be fabricated ensuring the drug loaded in the core layer can be released in a much slower manner. Our group recently loaded puerarin to reduce inflammation by micro-sol electrospinning (Fig. 7B). The drug release time reached as long as 40 days achieving a long-term endogenous inflammation suppressing effect. The successful abdominal wall reconstruction was attained due to the reduced early and long-term inflammatory response.

The unique porous interconnected 3D structure of electrospun fibrous scaffolds has always inspired bioengineers to design biomimetic wound healing materials. To overcome the quick degradation of biological tissue grafts and capitulate their excellent biocompatibility, biologically functional fibrous meshes have been developed by loading bioactive components (growth factors, peptides, proteins) and even cells (Fig. 7D). In a study by Dong et al., a novel electrospun composite scaffold was developed by culturing the rat adipose-derived stem cells (ADSCs), which can be induced into endothelial cells in vivo to enhance regeneration capacity when used in an abdominal wall defect [260]. The 3D porous structure with sufficient mechanical strength provided a high surface area and volume ratio for native tissue ingrowth while maintaining the structural integrity of the defect area. The thermosensitive component in this hybrid composite guaranteed the hydrophilicity and degradability of the biomimetic scaffold to avoid unnecessary foreign body reactions and promote cell adhesion and proliferation compared with the PLA group. Early vascularization was achieved by gene transfection using a lentiviral technique before the ADSCs were cultured on the fibrous scaffold. The cell-loaded biomimetic scaffold can hopefully replace the biological tissue grafts’ structural and functional superiorities, promising a beneficial treatment solution for abdominal wall hernia repair.

#### 5.2.4 Layer-by-layer electrospun hernia meshes

The biological tissue grafts reduced the adhesion formation to some extent thanks to their excellent biocompatibility—however, fast degradation and failure to provide mechanical support during the tissue remodeling process. Most biodegradable polymers can provide the required mechanical strength, but the instinctive inertness can cause foreign body reactions that end in abdominal organ adhesion. These consequences pushed researchers to rethink material design based on the abdominal wall's anatomical structure properties. The abdominal wall is anatomically composed of several layers, including the skin, subcutaneous fascia, muscle, and peritoneum. To achieve functional recovery, it is of great importance to consider the abdominal wall's structural differences, such as subcutaneous muscle and fascial tissue regeneration for resisting abdominal pressure and parietal peritoneum reconstruction, preventing internal organ adhesion, respectively. Given that, using multi-layered hernia meshes can replicate the structure and function of the abdominal wall and hopefully achieve a good outcome of abdominal wall reconstruction. Driven by such a design concept, Wang's team reported a double-layer nanofibrous scaffold consisting of polycaprolactone, graphene oxide, and chitosan using a consecutive electrospun approach (Fig. 7C) [319]. Graphene oxide can increase the composite scaffold's mechanical strength to simulate the abdominal wall's muscular layer, while the second later, chitosan, a common anti-adhesive component, simulates the peritoneal surface. Apart from the structural simulation, an FDA-approved drug was loaded to functionalize the composite for removing reactive oxygen species and neovascularization. Good cell viability confirmed the scaffold's cell-friendly character, while the in vivo histological studies demonstrated the reduced collagen deposition indicative of attenuated adhesion formation efficiency of chitosan. More importantly, newly formed blood vessels were signs of improved wound healing, posing a new horizon for abdominal wall defect repair.

The endless possibilities of electrospinning strategies have been adapted as an exceptional regenerative medicine platform providing cutting-edge drug delivery vehicles, tissue repair scaffolds, and dynamic monitoring systems while minimizing undesired side effects [[329], [334], [337]]. However, there is still a long way to go, to precisely mimic tissue structure and even create ex vivo organs for individual tissue regeneration. As for hernia repair, for example, the material choice with appropriate mechanical strength is essential while simultaneously improving the biocompatibility and regenerative capacity of the fiber meshes. Despite the endless efforts and satisfying results reported in electrospun hernia mesh fabrication, its limited practical applicability to promote the translating process due to lacking of more precise patient-oriented designing solutions. Besides, the structural difference between the electrospun fibrous meshes and commercial repair prosthetics have made the process more difficult. It is reasonable for surgeons to consider an engineered smart hernia mesh that has the mechanical strength and physical structure with the commercial meshes. Driven by this need, other tissue engineering approaches such as 3D printing technique have been explored in recent years.

### 5.3 3D printed scaffolds

So far, numerous meshes used for hernia repair made of both synthetic and biological materials have been presented in various shapes and compositions. However, there is no ideal mesh choice in the market possessing optimal tissue repair potential and mechanical performance while minimizing mesh-related side effects. On the other hand, the rise of the individualized treatment concept has pushed scholars to engineer a new generation of tissue repair materials to match individual organs and even with each patient. Consequently, intense efforts are still needed to engineer and develop cutting-edge meshes for hernia repair. 3D printing, or additive manufacturing, has come into the engineering world right under such high demands in the healthcare industry [338]. Patient-specific scaffold fabrication via layer-by-layer using 3D printing with the help of a computer-aided design approach is the main object of 3D printing.

**Fig. 8:**
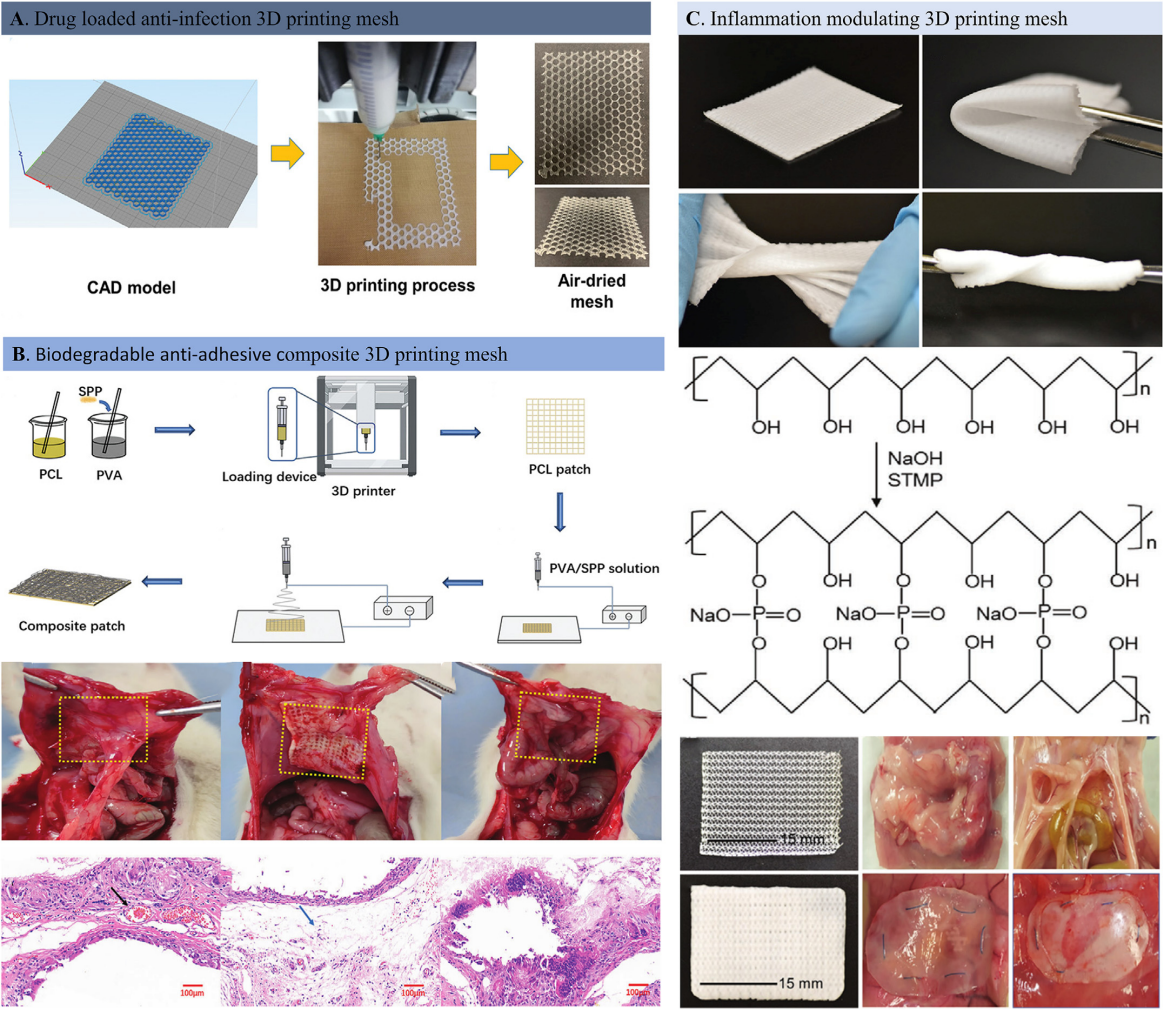
3D printing hernia meshes. A. 3D printing process of alginate and waterborne-polyurethane inks for fabricating hernia mesh implants with adequate morphological properties customizable to patient injury through computer-aided design model adaptation [343]. B. Schematic diagram of making a degradable 3D printing patch with an antiadhesive layer for hernia repair. Macropictures of hernia defect healing 4 weeks after patch implantation and HE staining microscope section [344]. C. Photographs of a 3D-printed bioscaffold composed of an in situ phosphate crosslinked poly (vinyl alcohol) polymer to capture the proinflammatory cytokines and chemokines to reduce the implant-related adhesion formation. The pictures of bioscaffolds retrieved after 2 and 4 weeks' implantation showed a much milder adhesion within the 3D printed hernia scaffold compared with the commercial PP one [345].

In recent years, innumerable 3D printed biomedical devices have been produced for tissue engineering, including but not limited to nerve, heart, skin, cartilage, and bone. Some experimental 3D printed devices have been approved by the US Food and Drug Administration for clinical use [[339], [340], [341]]. Besides, combined with other tissue engineering techniques, 3D printing scaffolds can be transformed into functional devices for potential individual treatment (Fig. 8A). Zhou et al. developed a tough and elastic hydrogel for soft tissue engineering using coaxial 3D printing with a special ink composed of catechol-modified hyaluronic acid and alginate [342]. Cell-scaffold interactions are necessary for tissue regeneration, which requires good cytocompatibility and cell adhesion properties. This study's multiple crosslinking strategies under mild conditions make it possible to integrate functional bioactive components that can promote cell adhesion and proliferation. Bio inks made of self-healing hydrogels as a cell carrier for tissue reconstruction are a new trend in regenerative medicine due to their steady rheological properties and post-printing crosslinking capabilities.

In a study conducted by Liu et al., a cell-laden 3D printed scaffold was developed using a self-healing hydrogel composed of injectable chitosan ink. Before 3D printing, the chitosan was functionalized by phenol to render an increased gelling rate, enhanced self-healing property, and post-printing crosslinking capability [346]. After secondary crosslinking, individually printed hydrogels can be set up into an organized construct broadening the tissue engineering application range of self-healing 3D printed scaffolds. Apart from the unique inks used for functional 3D printing, various 3D printing technology has been developed to satisfy various tissue regeneration requirements. One such example reported a new electrohydrodynamic 3D printing technology to fabricate ECM mimicking scaffolds with structural cues for specific cellular functions through electric force instead of laser, heat, or chemical reagents that will damage cells or tissue in varying degrees [347]. Incorporating the conductive component in this hybrid scaffold made it applicable for regenerating and repairing bio-conductive tissue in vivo, like myocardial, skeletal muscle, or nerve regeneration.

Just like other regenerative materials developed for tissue engineering, 3D printing is entitled to tailor a highly accurate patient-oriented biomedical material. This state-of-the-art technology aids in developing more complex and precise devices that cannot be fabricated by conventional techniques with easier modifiability. Gu's group reported a smart hernia repair mesh composed of a PCL 3D printing scaffold and collagen electrospinning membrane [348]. The interconnection between macro-and micro-fiber with 3D porous architecture promoted cellular entrapment with more suitable filament spacing. Besides, collagen, a critical ECM component, is a cell-friendly protein broadly used in tissue engineering. The newly formed blood vessels and rapidly integrated regenerative cells indicated the biocompatibility of this scaffold rendered by the collagen component. In another case study, Danker et al. reported that a recurrent chest/abdominal wall composite hernia was reconstructed using a 3D-printed titanium plate [349]. In such an extensive defect scenario, patient-oriented reconstruction was needed with structural and functional integrity, which can be achieved by 3D printing technology. The reported case demonstrated a durable hernia reconstruction of as long as 1.5 years without recurrence and other complications, making this convenient and practical surgical device inspiring for future surgical treatments.

Post-implantation adhesions have been the main obstacle in plastic surgery for decades, and numerous strategies were proposed with the joint efforts of engineers and surgeons. Despite the variety of anti-adhesive solutions, there isn't an effective one yet. However, scholars did not stop exploring new ways of preventing adhesion; on the contrary, they kept searching for solutions by looking deeply into the mechanism of visceral adhesion. Most surgical operation within the peritoneal cavity triggers an intense inflammatory reaction caused both by the implant-related foreign body reaction and destruction of the peritoneal protective layer, which will expose the cavity organs to an unregulated inflammatory process, eventually ending in adhesion formation [350]. The persistent inflammatory reaction can be related to the inflammatory cells, which are attracted to the damage site by cytokines and chemokines and secrete these chemo-attractive factors, consequently inducing a vicious circle hindering the repair process as some of such inflammatory factors are charged molecules that an oppositely charged material can capture through electrostatic interactions. Based on the mechanism of adhesion formation, Shin et al. designed a 3D printing scaffold to modulate excessive inflammation for hernia repair (Fig. 8C) [345]. In this study, a negatively charged 3D scaffold was obtained by printing the polymer poly (vinyl alcohol) (PVA), which was in situ crosslinked by negatively charged phosphate under mild conditions. Apart from the ideal mechanical strength that perfectly matches the abdominal pressure, one of the most impressive properties of this biofabric was the negative charges that allow it to capture the positively charged proinflammatory cytokines and chemokines. Owing to its inherent noninflammatory character and excellent mechanical force, the 3D printed hernia repair scaffold can potentially minimize mesh-related adhesions and improve abdominal wall repair outcomes. Another anti-adhesion approach reported by researchers is a double-layer patch with different functions on each side. For example, in a recent study conducted by Hu et al., a Janus structure was developed by combining 3D printing and electrospinning technology (Fig. 8B) [351]. The 3D PCL scaffold provided the fundamental mechanical support for abdominal contents, while the electrospun layer made of PVAand soybean peptide served as an anti-adhesive barrier between the peritoneum and visceral organs. By separating the mechanical supportive and anti-adhesive parts, the composite scaffold's repair effect on abdominal wall hernia reached the highest degree as the cytocompatibility and degradability provided the application safety. In vitro results showed that the 3D printed layer promoted human umbilical vein endothelial cell proliferation, while the electrospun presented anti-adhesive properties characterized by in vivo collagen capsule thickness. Overall, the experimental results in this study confirmed the double-layer structure's promising potential for adhesion-free hernia repair.

To sum up, 3D printing technology facilitates a wide variety of highly customized and even patient-oriented biocompatible scaffolds that can be further functionalized by combining distinct structures and biological contents [352,353]. Although 3D printing has already made its way into clinics, there are still challenges that need to be conquered in the future [354]. For instance, for abdominal wall reconstruction, the heterogenous structure of different muscle layers requires more precise inventory printing techniques and tissue engineering capabilities. Besides, direct contact with the abdominal cavity fluid may accelerate the degradation of implanted scaffolds, making it necessary to consider sustainability when designing 3D printed fabricates for abdominal wall defect reconstruction. Despite the shortcomings, 3D printing technology constantly evolves to stride forward into operating rooms without delay.

## 6 Challenges and future perspectives

With the development of basic surgical theory and surgical techniques, understanding of human anatomy, progress in bio-functional materials, modern hernia repair has made rapid progress. The concept of “tension-free repair” was proposed in 1980s, bringing hernia surgery into a new era. Since then, the principles of modern surgery advocated using prosthetic materials to repair hernia defects, so as to save lives, improve treatment effects, and improve quality of life. Although huge progress has been made in recent decades in tension-free hernia repair using various prosthetics such as absorbable, non-absorbable or partially absorbable with different functionalities, and the tissue engineering smart hernia meshes made by different approaches, there are still unsolved challenges that must be taken into consideration to achieve the ultimate goal of replacing the like with like. The challenges and future perspectives in engineering science for hernia repair materials are summarized as follows:

1) The focus in hernia repair has been put on the development of ideal hernia materials over the past decades. However, as the precise treatment concept has entered into the clinical practice, it is of great importance to pay attention to the prevention side of the diseases. Although hernia has been viewed as a pure surgical problem that caused by the local weak areas in the abdomen, the findings made in basic research shed light on its molecular developing mechanisms. Recent evidence showed there is decrease of certain microelement in hernia sites such as copper and zinc. This indicates that it’s of great importance to take actions to screen relevant indicators and design some smart materials before its occurrence. This is a future direction for both the clinicians and engineers to develop practical devices that can either precisely screen or regulate hernia indicators.
2) As biomaterial science begins to enter the stage of precise regulation through disease mechanisms, more efforts need to be paid to unveil the biological basis of hernia development and even hernia recurrence. For instance, there are certain genes, proteins and enzymes that regulate the process that can be intervened by well-designed engineering materials. For example, the lysis oxidase (LOX) family of enzymes that participate in collagen and elastin crosslinking are important regulative factors during the ECM maturing process. Any abnormalities in LOX can result in ECM-related diseases like pelvic organ prolapse and abdominal wall hernia. Besides, the recent progress in single-cell RNA sequencing, metabolomic analysis, and bioinformatics analysis can help researchers to deepen the understanding of the unveiled mechanism of abdominal wall hernia. Given the fact, smart meshes can be designed to upregulate or downregulate the key molecules to achieve more effective and precise treatment of hernia. In addition, different smart materials can be designed according to the hernia classification because of the different development mechanisms.
3) A more effective evaluation system on the clinical applicability, safety, and biocompatibility is expected both before designing and premarketing hernia repair meshes. The evaluation methods published works regarding hernia repair mainly includes three aspects: characterization of prepared materials; in vitro biocompatibility experiments; in vivo reconstructive efficacy evaluation. Although the approaches of material characterization and cell compatibility analysis relatively centralized, the in vivo evaluation system changes between countries and even research groups. For example, some studies report the collagen deposition as an indicative of successful reconstruction in animal studies as the defect area filled with newly generated collagen tissue. However, in other research results, the collagen is considered to be the marker of fibrous tissue which is caused by inflammation. There has not a unified standard for animal study results to make further improvements to the existing strategies.
4) The engineered hernia materials that have been developed are mostly limited to the traditional tissue engineering techniques. Nonetheless, the future hernia repair materials should be even more intelligent by combining photosensitive, electro-magnetic responsive, sonodynamic and mechanosensitive components to on-demand control the hernia materials rather than passively reconstruct the damaged tissues. Advanced biotechnologies need to be developed to improve host tissue response and mesh integration, along with improved manufacturing to make custom hernia meshes to meet the desired demand of precise treatment.

## 7 Conclusion

Myriad materials and techniques correlated with various approaches have been adopted to functionalize hernia repair meshes. The scientific advances in this research field have been enormous and it became clear that tissue engineering approaches reported to date are only the tip of the iceberg. Based on the evaluation of safety and effectiveness, the modern era of hernia repair mesh keeps evolving towards the improvement of patient care.

Up-to-date, polypropylene hernia repair mesh is believed to be the most frequently used porous polymer construct in clinical practice owing to its good physicochemical and biological properties and cost-effectiveness. However, unexpected surgical complications include mesh-related adhesion, pain, erosion, and infection. To overcome these clinical complications, surgeons and engineers have proposed a series of hernia repair strategies throughout these decades. In this paper, we reviewed the history of hernia repair meshes that evolved from synthetic non-absorbable protheses, absorbable synthetic materials, biological grafts, partially absorbable engineered polypropylene meshes to smart hernia reconstructive scaffolds, such as hydrogel scaffolds, electrospun fibers, and 3D printing patches with the hope of fabricating precise, customized, and optimal hernia repair materials. The effects regarding to their functionalization with advantages and disadvantages were also discussed systemically. Despite a long history of evolution and great advances made in hernia mesh designing, significant challenges remain, and the exploration of novel materials and designs are still critical to improving abdominal wall reconstruction in the future.

## Declaration of competing interest

The authors declare that they have no known competing financial interests or personal relationships that could have appeared to influence the work reported in this paper.

## Data Availability

Data will be made available on request.
